# Clonal heterogeneity and rates of specific chromosome gains are risk predictors in childhood high‐hyperdiploid B‐cell acute lymphoblastic leukemia

**DOI:** 10.1002/1878-0261.13276

**Published:** 2022-07-19

**Authors:** Mireia Ramos‐Muntada, Juan L. Trincado, Joan Blanco, Clara Bueno, Virginia C. Rodríguez‐Cortez, Alex Bataller, Belén López‐Millán, Claire Schwab, Margarita Ortega, Pablo Velasco, Maria L. Blanco, Josep Nomdedeu, Manuel Ramírez‐Orellana, Alfredo Minguela, Jose L. Fuster, Esther Cuatrecasas, Mireia Camós, Paola Ballerini, Gabriele Escherich, Judith Boer, Monique DenBoer, Jesús M. Hernández‐Rivas, Maria J. Calasanz, Giovanni Cazzaniga, Christine J. Harrison, Pablo Menéndez, Oscar Molina

**Affiliations:** ^1^ Cell Biology, Physiology and Immunology Department, Genetics of Male Fertility Group Universitat Autònoma de Barcelona Spain; ^2^ Department of Biomedicine, School of Medicine, Josep Carreras Leukemia Research Institute University of Barcelona Spain; ^3^ CNAG‐CRG, Centre for Genomic Regulation (CRG) Barcelona Institute of Science and Technology (BIST) Spain; ^4^ Red Española de Terápias Avanzadas (TERAV) ISCIII Barcelona Spain; ^5^ Centro de Investigación Biomédica en Red de Cáncer (CIBER‐ONC) ISCIII Barcelona Spain; ^6^ Hematology Department, Hospital Clínic de Barcelona, IDIBAPS University of Barcelona Spain; ^7^ Wolfson Childhood Cancer Research Centre Newcastle University UK; ^8^ Hematology Service, Experimental Hematology, Vall d'Hebrón Institute of Oncology (VHIO) Vall d'Hebrón Hospital Universitari Barcelona Spain; ^9^ Pediatric Oncology and Hematology Department Vall d'Hebrón Hospital Barcelona Spain; ^10^ Hematology Laboratory Hospital Sant Pau Barcelona Spain; ^11^ Hematology Diagnostic Laboratory Hospital Niño Jesús Madrid Spain; ^12^ Immunology Service Clinic University Hospital Virgen de la Arrixaca and Instituto Murciano de Investigación Biomédica (IMIB) Murcia Spain; ^13^ Pediatric Hematology and Oncology Department Hospital Clínico Universitario Virgen de la Arrixaca, Instituto Murciano de Investigación Biosanitaria (IMIB) Murcia Spain; ^14^ Hematology Laboratory Institut de Recerca Hospital Sant Joan de Déu Barcelona Spain; ^15^ Hematology Laboratory, Hospital Sant Joan de Déu University of Barcelona Spain; ^16^ Leukemia and Other Pediatric Hemopathies, Developmental Tumor Biology Group Institut de Recerca Hospital Sant Joan de Déu Barcelona Spain; ^17^ Centro de Investigación Biomédica en Red de Enfermedades Raras (CIBERER) Instituto de Salud Carlos III Madrid Spain; ^18^ AP‐HP, Service d'Hématologie Pédiatrique Hôpital A. Trousseau Paris France; ^19^ Department of Pediatric Hematology and Oncology University Medical Center Hamburg Germany; ^20^ Princess Máxima Center for Pediatric Oncology Utrecht The Netherlands; ^21^ Oncode Institute Utrecht The Netherlands; ^22^ Department of Pediatric Oncology/Hematology Erasmus MC – Sophia Children's Hospital Rotterdam The Netherlands; ^23^ Departamento de Hematología, Salamanca‐IBSAL Hospital Universitario de Salamanca Spain; ^24^ CIMA Lab Diagnostics Universidad de Navarra Pamplona Spain; ^25^ Fondazione Tettamanti Monza Italy; ^26^ Institució Catalana de Recerca i Estudis Avançats (ICREA) Barcelona Spain

**Keywords:** chromosomal gains, clonal heterogeneity, computational modeling, high‐hyperdiploid B‐ALL, risk predictors, sequential iFISH

## Abstract

B‐cell acute lymphoblastic leukemia (B‐ALL) is the commonest childhood cancer. High hyperdiploidy (HHD) identifies the most frequent cytogenetic subgroup in childhood B‐ALL. Although hyperdiploidy represents an important prognostic factor in childhood B‐ALL, the specific chromosome gains with prognostic value in HHD‐B‐ALL remain controversial, and the current knowledge about the hierarchy of chromosome gains, clonal heterogeneity and chromosomal instability in HHD‐B‐ALL remains very limited. We applied automated sequential‐iFISH coupled with single‐cell computational modeling to identify the specific chromosomal gains of the eight typically gained chromosomes in a large cohort of 72 primary diagnostic (DX, *n* = 62) and matched relapse (REL, *n* = 10) samples from HHD‐B‐ALL patients with either favorable or unfavorable clinical outcome in order to characterize the clonal heterogeneity, specific chromosome gains and clonal evolution. Our data show a high degree of clonal heterogeneity and a hierarchical order of chromosome gains in DX samples of HHD‐B‐ALL. The rates of specific chromosome gains and clonal heterogeneity found in DX samples differ between HHD‐B‐ALL patients with favorable or unfavorable clinical outcome. In fact, our comprehensive analyses at DX using a computationally defined risk predictor revealed low levels of trisomies +18+10 and low levels of clonal heterogeneity as robust relapse risk factors in minimal residual disease (MRD)‐negative childhood HHD‐B‐ALL patients: relapse‐free survival beyond 5 years: 22.1% versus 87.9%, *P* < 0.0001 and 33.3% versus 80%, *P* < 0.0001, respectively. Moreover, longitudinal analysis of matched DX‐REL HHD‐B‐ALL samples revealed distinct patterns of clonal evolution at relapse. Our study offers a reliable prognostic sub‐stratification of pediatric MRD‐negative HHD‐B‐ALL patients.

## Graphical abstract

Automated sequential‐iFISH coupled with single‐cell computational modeling in high‐hyperdiploid (HHD) B‐ALL samples revealed low levels of trisomies 18, 10 and clonal heterogeneity at diagnosis as robust predictive risk factors, properly classifying ~ 85% of patients according to relapse risk. We propose a simple and reliable protocol for improving patient stratification at diagnosis easily applicable into the clinical routine of hematoncology laboratories.

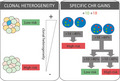

**Abbreviations**
B‐ALL: B‐cell acute lymphoblastic leukemia
BM: bone marrow
CIN: chromosome instability
CR: complete remission
DX: diagnostic
EFS: event‐free survival
HHD: high‐hyperdiploidy
HSPC: hematological stem/progenitor cells
iFISH: interphase fluorescence *in situ* hybridization
MN: modal number
MRD: minimal residual disease
OS: overall survival
PMC: percentage of major clone
R/R: refractory/relapse
REL: relapse
RFS: relapse‐free survival
Seq‐iFISH: sequential interphase fluorescence *in situ* hybridization

## 1 Introduction

B‐cell acute lymphoblastic leukemia (B‐ALL) is the most common cancer in children, characterized by the accumulation of B‐cell progenitors in the bone marrow (BM) [1]. Over the past 20 years, much progress has been made in understanding the biology of the disease providing significant progress in molecular diagnosis and risk stratification for treatment, leading to considerable improvements in disease management and clinical outcome [2, 3, 4]. Despite these encouraging advances, the outcome of patients with refractory/relapsed (R/R) B‐ALL remains dismal, resulting in the most common cause of death from malignancy in children [5, 6, 7].

Aneuploidy has long been considered a significant prognostic factor in childhood B‐ALL, with early studies suggesting that B‐ALL with chromosomal gains (hyperdiploidy) is associated with a favorable outcome [8]. Subsequent studies refined the prognostic value of hyperdiploid B‐ALL, demonstrating that patients with a modal chromosome number (MN) of > 50 (high hyperdiploidy; HHD) had the most favorable outcome [9, 10]. Indeed, HHD defines the most frequent cytogenetic subgroup in childhood B‐ALL, accounting for ~ 30% of cases, and is typically associated with favorable prognostic features [11]. Typically, children with HHD‐B‐ALL achieve negative minimal residual disease (MRD) after induction treatment and have excellent cure rates, with 5‐year event‐free survival (EFS) and overall survival (OS) rates of ~ 75%, and ~ 90%, respectively [11].

The distribution of chromosomal gains in HHD‐B‐ALL is non‐random, with gains of chromosomes X, 4, 6, 10, 14, 17, 18, and 21 being the most frequent [12]. Despite chromosomal gains *per se* being accepted as a prognostic factor, specific trisomies have been implicated as better indicators of outcome than MN. The Pediatric Oncology Group showed that trisomies 4 and 10 were associated with a good prognosis [13]. Similarly, the Children's Cancer Group reported that trisomies 10 and 17 conferred a superior outcome in HHD‐B‐ALL [10], while the Children's Oncology Group and others identified that combined trisomies 4, 10, and 17 were the strongest indicator of favorable outcome in HHD‐B‐ALL [14, 15, 16]. Moreover, Moorman et al. showed superior outcomes for HHD‐B‐ALL patients with trisomies 4, 10, or 18, with trisomy 18, together with patient age, being the strongest independent prognostic indicator [17, 18]. Overall, although specific trisomies have been associated with survival, the exact combination of chromosomal gains with the highest and most reliable predictive value remains inconclusive.

Cytogenetic analysis of metaphase chromosomes has been the gold standard technique used to assess the prognostic value of chromosomal gains in HHD‐B‐ALL [19, 20]. However, it has several limitations hampering investigation of the genomic heterogeneity and complexity of the disease, including: (a) low number of metaphases for analysis, due to failure of blast growth *ex vivo*, (b) biased selection of clones through cell culture, and (c) poor chromosome quality, especially in HHD‐B‐ALL, preventing accurate assessment of chromosome number and identity due to chromosome condensation defects [21]. Thus, fluorescence *in situ* hybridization on interphase nuclei (iFISH) in combination with conventional cytogenetics has proved to be useful for the accurate detection of chromosomal gains, as iFISH does not require dividing cells and it can detect hidden clones and mosaicism within the samples [22]. Indeed, the use of iFISH by independent groups has revealed high levels of clonal heterogeneity in HHD‐B‐ALL samples [23, 24, 25], with multiple subclones containing different combinations of chromosomal gains in individual cells, suggesting chromosomal instability (CIN) within HHD‐B‐ALL. Notably, sequential iFISH analysis (seq‐iFISH), which permits identification of the eight typically gained chromosomes in HHD‐B‐ALL, showed changes in chromosome number to be hierarchical with sequential chromosomal gains, rather than losses, retained from lower to higher MNs [25]. Notably, while CIN has been proposed as an underlying mechanism in HHD‐B‐ALL, its association with patient outcome has not been investigated so far.

In this study, we sought to investigate the potential prognostic impact of clonal heterogeneity in HHD‐B‐ALL. To this end, we investigated the presence of eight typically gained chromosomes in HHD‐B‐ALL patients. We used automated seq‐iFISH coupled with single‐cell computational modeling in individual cells from a large cohort of primary samples obtained at disease presentation (diagnostic, DX). HHD‐B‐ALL patients were either in complete remission (CR), disease‐free and without relapse after a minimum of 5 years after treatment, or have relapsed (REL) within this timeframe. In addition, we performed longitudinal analyses to study clonal evolution during disease progression within matched DX‐REL HHD‐B‐ALL samples. Our data showed a high degree of clonal heterogeneity and hierarchical chromosomal gains in DX samples and revealed specific chromosomal gains and clonal heterogeneity to be potent predictors of relapse in HHD‐B‐ALL patients who were MRD‐negative after induction treatment.

## 2 Material and methods

### 2.1 Patient and donor samples

**Fig. 1:**
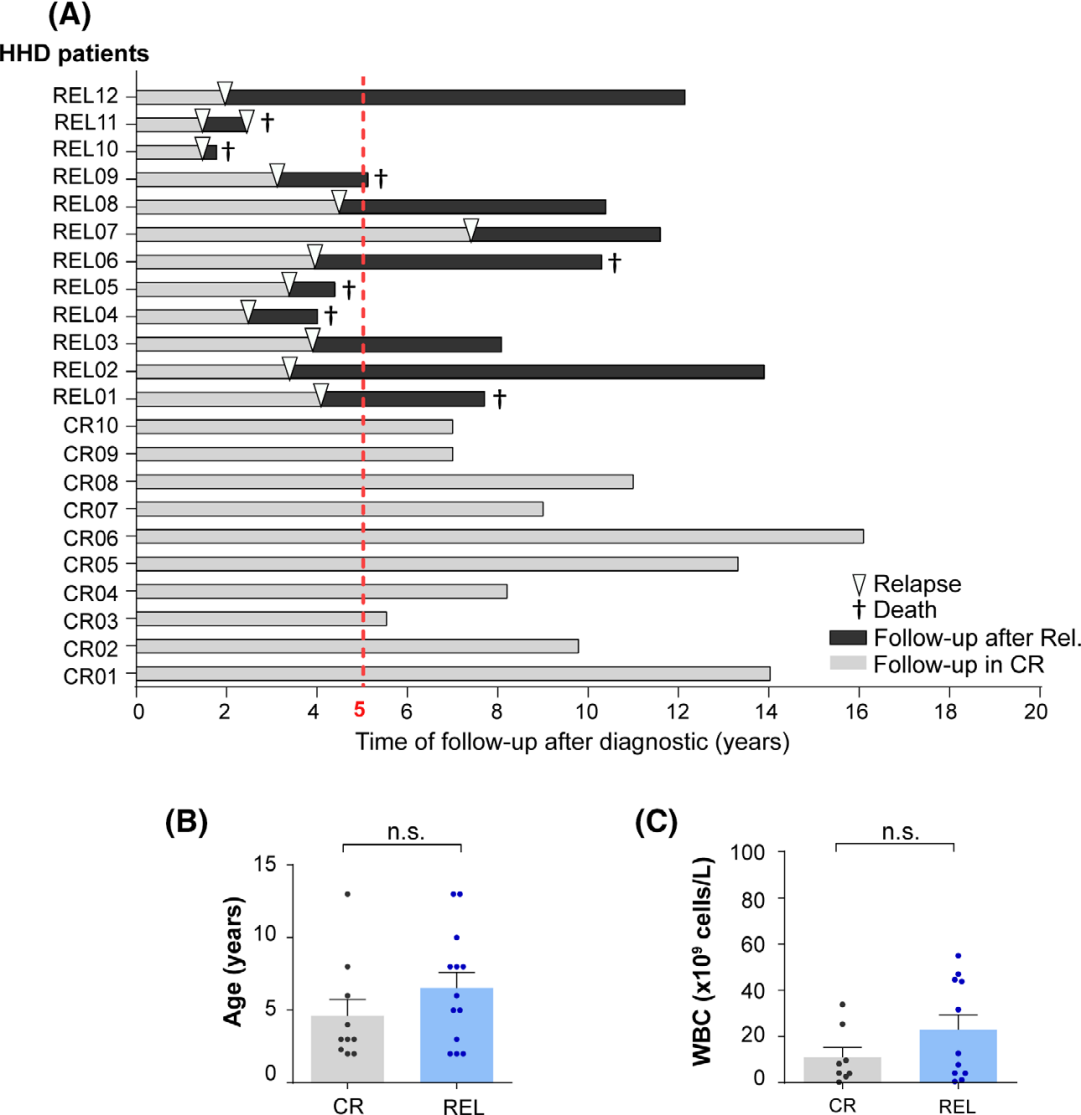
Clinical outcome and biological features of the high‐hyperdiploid B‐cell acute lymphoblastic leukemia (HHD‐B‐ALL) patients analyzed by seq‐iFISH. (A) Time of follow‐up after diagnosis (DX) of the HHD‐B‐ALL patients analyzed by seq‐iFISH. Complete remission (CR) denotes those patients who remained disease‐free after a minimum of 5 years of follow‐up after treatment denoted as a dashed red line (*n* = 10). Relapse (REL) denotes those patients who relapsed within this timeframe (except one patient who relapsed after 7 years; *n* = 12). Light and dark gray bars represent follow‐up in CR and after relapse, respectively. Arrow heads depict the time of relapse. † denotes patient's death. (B,C) age (B) and WBC (C) of CR and REL patients. Bars represent the mean values of each group and the error bars represent the standard error of the mean (SEM). Each dot represents an individual patient. Two‐sided unpaired *t*‐test. [Colour figure can be viewed at wileyonlinelibrary.com]

B‐ALL diagnosis was based on French‐American‐British and World Health Organization classifications. BM samples (*n* = 82) from pediatric patients with confirmed HHD‐B‐ALL were obtained from collaborating hospitals. A number of samples (*n* = 32; discovery cohort) were available for seq‐iFISH and computational modeling (Table 1). Among these samples were DX samples (*n* = 22) obtained at disease presentation from patients who either remained alive in CR, without relapses after a minimum of 5‐year follow‐up (CR, *n* = 10), or who relapsed within this timeframe (except one patient who relapsed after 7 years; REL, *n* = 12). The remaining samples (*n* = 10) were relapse samples with matched corresponding DX samples (Table 1, Fig. 1). An independent cohort of 50 DX HHD‐B‐ALL samples (validation cohort) was used for blind validation of computational modeling data (Table S1). Fetal liver (FL) CD34^+^ hematopoietic/stem progenitor cells (HSPC; *n* = 2) and peripheral blood mononuclear cells (PBMNC), obtained from a healthy donor, were used as technical controls. Fetal tissue was collected from the Medical Research Council (MRC)/Wellcome Trust Human Developmental Biology Resource with informed consent and approval by our local ethics committee.

**Table 1:** Cytogenetic and clinical data of all the childhood HHD‐B‐ALL samples used for seq‐iFISH analyses. DX, Diagnosis; RX: Relapse. CR: patients who remained disease‐free after a minimum of 5 years of follow‐up after treatment; REL: patients who relapsed within this timeframe (except REL07, relapse after 7 years).

Code	Event	Karyotype	Age	Gender	% blasts^a^	WBC dx (×10^9^·L^−1^)	Follow‐up^b^ ^,^ ^c^	Treatment protocol	Relapse	Exitus
CR01	Dx	58,XY,+X,−Y,+4,+6,+8,+10,+11,+12,+14,+17,+18,+21,+21,+22 (SNP6)	3	M	91.6	195	14	COALL03‐HR‐S	No	No
CR02	Dx	53,XX,+X,+X,+6,+14,+17,+21,+21 (SNP6)	2	F	89.2	9	9.6	COALL03LR‐R	No	No
CR03	Dx	55 XXYY,+4,+6,+12,+15,+18,+21	8	M	98.6	‐	5.5	SEHOP/PETHEM2013	No	No
CR04	Dx	54,XX,+X,+6,+8,+14,+17,+18,+21,+21[15]/54,idem,−13,+mar[11]/46,XX[19]	4	F	89	8.2	8.2	ALL10‐MR	No	No
CR05	Dx	57,XX,+X,+X,+4,+6,+8,+10,+14,+17,+18,+21,+21 (SNP6)	2	F	99	4	13.3	COALL03‐LR‐R	No	No
CR06	Dx	54,X,+X,Y,+6,+10,+14,+17,+18,+21,+mar[30]	3	M	94.2	33.9	16	PETHEMA LAL96	No	No
CR07	Dx	55,XXY,+3,+4,+6,+10,+14,+18,+21,+21/46,XY	2.3	M	93.9	25.4	9	SHOP‐2005	No	No
CR08	Dx	60,XY,+X,+4,+6,+7,+8,+9,+14,+17,+18,+21,+21,+3mar/46,XY	6	M	82.4	2.67	11	SHOP‐2005	No	No
CR09	Dx	57,XXY,+4,+6,+8,+10,+10?,+13,+14,+17,+18,+21[14]/46,XY[16]	13	M	90.1	4.2	7	SHOP‐2005	No	No
CR10	Dx	54,XXX,+4,+6,+8,inv(9)(p11q12),+14,+17,+18,+21[22]/46,XX,inv(9)(p11q12)[2]	3	F	95.6	9.7	7	SHOP‐2005	No	No
		Mean ± SEM	4.6 ± 1.1	6/4		31.4 ± 19.7	10.1 ± 1.1			
REL01	Dx	59,XY,+?X,+4,+6,+7,+8,+12,+14,+?add(17)(p1),+18,+18,+21,+der(?)t(?;5)(?;q?13),+mar[6]/63,idem,dup(1)(q2q3),+22,inc[7]/46,XY[5]	6	M	80.6	4.2	4.4	UKALL2003‐SR	Yes	Yes
	Rx	55‐57,X,−Y,+X,+8,+?10,+12,+14,+?add(17)(p1),+18,+21,+der(?)t(?;5)(?;q?13),inc[cp7]/46,XY[25]								
REL02	Dx	51,XX,+X,+8,+14,+21,+21[5]/46,XX[9]	2	F	95.9	119	3.6	UKALL2003‐B	Yes	No
	Rx	51,XX,+X,+8,del(11)(q2?2),+14,+21,+21[12]/46,XX[8]								
REL03	Dx	54,XY,+X,+4,+6,+10,+14,+add(19)(p13),+20,+21[cp8]	13	M	98	1.2	4.2	UKALL2003‐B	Yes	No
	Rx	54 ~ 55,XY,+X,+4,+5,+6,+10,+14,add(19)(p13),? + 20,+21,+21,+mar[cp9]/46,XY[1]								
REL04	Dx	46,XX[20]/High hyperdiploid by FISH	13	F	72.9	4.1	2.6	UKALL2003‐A	Yes	Yes
	Rx	55,XX,+X,+1,+4,+8,+14,+15,+21,+21,+mar[cp4]/46,XX[16]								
REL05	Dx	52‐56,XY,+X,dup(1)(q25q44),+4,+8,+10,+11,+14,+18,+21,+21,+mar[cp7]/46,XY[3]	8	M	96.1	47	3.5	UKALL2003‐A	Yes	Yes
	Rx	High hyperdiploid by FISH								
REL06	Dx	46,XY[20]/High hyperdiploid by FISH	8	M	50.2	7.8	4.2	UKALL2003‐A	Yes	Yes
	Rx	High hyperdiploid by FISH								
REL07	Dx	56,XY,+X,ins(1;?),(q21.3;?),+4,+6,+14,+17,+18,+21,+21,+22,+mar[5]/46,XY[5]	2	M	86.5	55	7.2	UKALL2003‐C	Yes	No
	Rx	46,XY[30]/High hyperdiploid by FISH								
REL08	Dx	High hyperdiploid by FISH	8	M	79.3	44.6	4.6	UKALL2003‐A	Yes	No
	Rx	52,XY,+X,+Y,+14,+21,+21[1]/46,XY[9]								
REL09	Dx	51,XX,+X,+6,+14,+21,+21[16]/51,idem,add(9)(q34)[2]/46,XX[2]	3	F	77.2	31.7	3.3	UKALL2003‐A	Yes	Yes
	Rx	52,XX,+X,+6,+14,+17,+21,+21[13]/46,XX[3]								
REL10	Dx	53,XX,+4,+6,+14,+17,+18,+2mar[8]/46,XX[22]	2	F	71.1	43.9	2	PETHEMA LAL 96	Yes	Yes
	Rx	53,XX,+4,+6,+14,+17,+18,+2mar[36%]/46,XX[64%]								
REL11	Dx	59,XXY,+Y,der(1)(q?),+4,+5,+6,+8,+10,+11,+18,+18,+21,+22,mar[9]/46,XY[41]	5	M	34.7	0.54	2	LAL‐SHOP‐2005	Yes	Yes
REL12	Dx	57,XY,der(1)(p?q?),+4,10mar[56%]/46,XY[44%]	5	M	65.5		0.5	PETHEMA LAL 96	Yes	No
		Mean ± SEM	6.5 ± 1.1	8/5		32.6 ± 9.9	3.5 ± 0.5			

^a^ As observed by FISH (% HHD cells).

^b^ Indicates follow‐up in CR for non‐relapsing patients.

^c^ Indicates time‐to‐relapse for relapsing patients.

The study was approved by the Barcelona Clínic Hospital Institutional‐Review Ethics Board (HCB/2014/0687). The study methodologies conformed to the standards set by the Declaration of Helsinki. The experiments were undertaken with the understanding and written consent of each subject.

### 2.2 Sequential fluorescence *in situ* hybridization

**Fig. 2:**
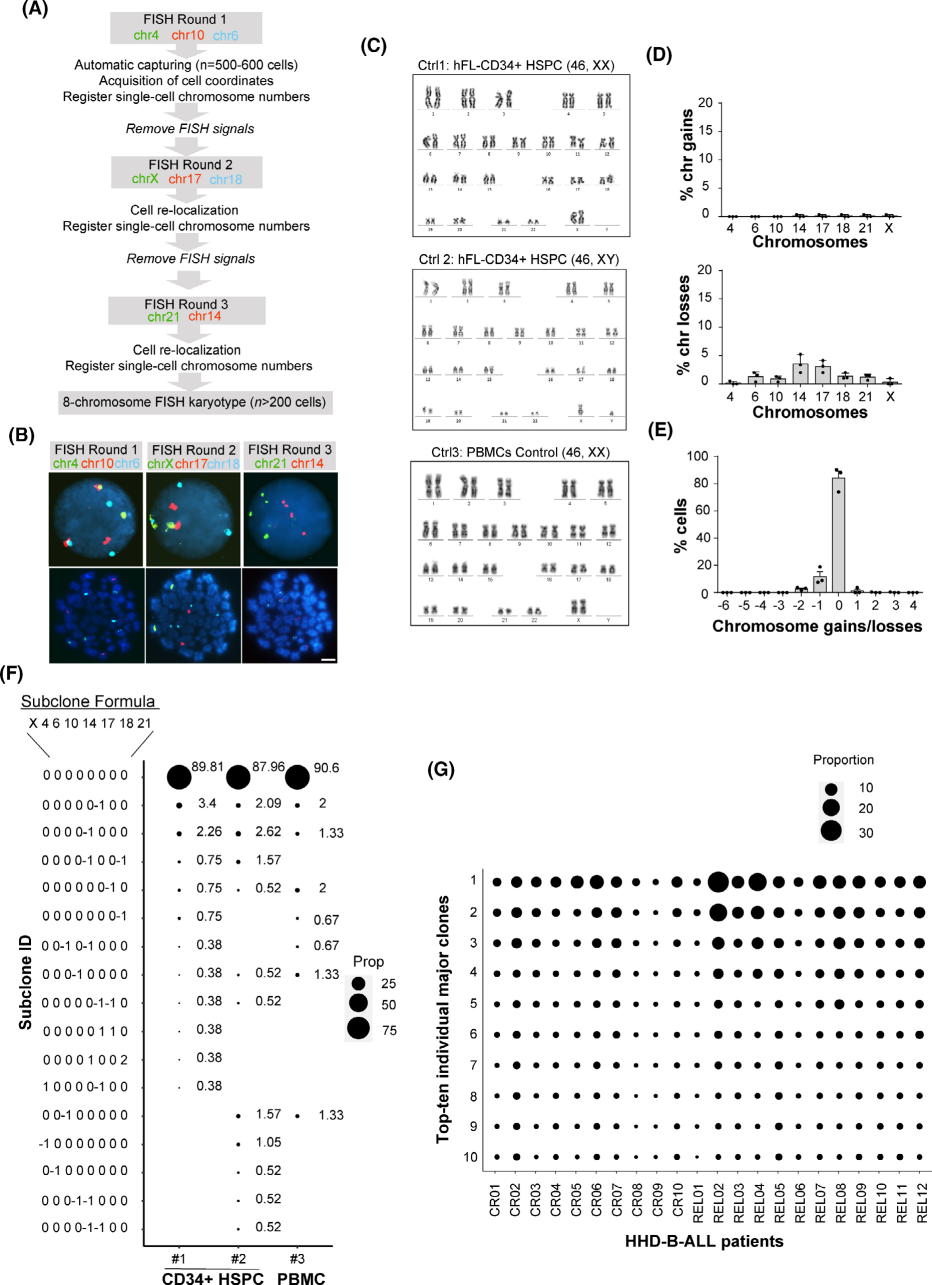
Reliability of the seq‐iFISH analyses employed for detection of clonal heterogeneity in high‐hyperdiploid B‐cell acute lymphoblastic leukemia (HHD‐B‐ALL). (A) Scheme depicting the seq‐iFISH stepwise analysis. (B) Representative images of consecutive FISH rounds using the indicated chromosomes in the same cell. Top and bottom panels show interphase and metaphase cells, respectively. Scale bar = 10 μm. (C) Karyotypes of the indicated control (ctrl) samples; *n* = 20 metaphases per sample. (D) Frequency of chromosomal gains (*top*) and losses (*bottom*) observed for each chromosome by seq‐iFISH analysis in control samples. (E) Modal number (MN) of chromosomal gains/losses in control samples. Graphs represent the mean value of three independent experiments and error bars represent the SEM; *n* = 200 nuclei were analyzed per experiment. (F) Single‐cell analysis showing the size of diploid and ‘false aneuploid’ clones observed in the control samples. Subclone formulas/codes on the left indicate the number of gains (1 or 2) or losses (−1) for the chromosomes X, 4, 6, 10, 14, 17, 18, and 21, respectively; *n* = 150 nuclei, 265 nuclei and 191 nuclei for controls 1, 2, and 3, respectively. (G) Single‐cell analysis showing the 10 major clones observed at diagnosis (DX) in all HHD‐B‐ALL patients; *n* = a minimum of 200 nuclei per sample. The size of the black circles in (F and G) represents the proportion of cells with the indicated subclone in each sample. [Colour figure can be viewed at wileyonlinelibrary.com]

Seq‐iFISH analyses were performed on cell suspensions from HHD‐B‐ALL BM samples fixed in methanol : acetic acid (3 : 1). Three successive hybridization rounds were performed on the same sample to assess the presence of the eight chromosomes typically gained in HHD‐B‐ALL (Fig. 2A,B). In the first hybridization round, triple‐color FISH was performed with centromere enumeration probes (CEP; Abbott Molecular Inc., Des Plaines, IL, USA) for chromosomes 4 (4p11‐q11 alpha‐satellite DNA; *Spectrum Green*), 6 (D6Z1; *Spectrum Aqua*), and 10 (10p11.1–q11.1 alpha‐satellite DNA; *Spectrum Orange*). In the second round, triple‐color FISH was performed with CEP probes for chromosomes X (DXZ1; *Spectrum Green*), 17 (D17Z1; *Spectrum Orange*) and 18 (D18Z1; *Spectrum Aqua*). In the third round, dual‐color FISH was performed with a locus‐specific probe for chromosome 21 (21q21.1, 5‐Fluorescein; Empire Genomics) and a subtelomeric probe for chromosome 14 (D14S1420; *Spectrum Orange*; Abbott Molecular Inc.). Seq‐iFISH was performed following standard procedures [26, 27]. In brief, slides were dehydrated in an ascending ethanol series and denatured in 70% formamide/2 × SSC at 73 °C for 2 min. DNA probes mixed in hybridization buffer (Abbott Molecular Inc.) were denatured at 73 °C for 5 min before hybridization in a humid chamber at 37 °C overnight. Slides were then washed twice in 0.4 × SSC containing 0.03% NP40 at 73 °C and then in 2 × SSC at room temperature for 2 min. Slides were mounted with 4′,6‐diamino‐2‐phenylindole (DAPI) solution for DNA counterstaining. DNA probes were removed between hybridization rounds by washing the slides in 0.0625 × SSC and incubating at 73 °C for 5 min.

### 2.3 Fluorescence microscopy and nuclei re‐localization

Automated FISH evaluation was performed using an Olympus BX‐61 epifluorescence microscope coupled to spot ax software (Applied Imaging, Newcastle, UK), and equipped with a BX‐UCB motorized stage (Olympus), a 60× objective and narrow band‐pass fluorescence filters specific for DAPI, Aqua, fluorescein isothiocyanate (FITC), and cyanine 3 (Cy3). After the first hybridization round, slides were scanned using nuclei recognition morphometric parameters and FISH signal capture [28]. Merged images (overlay of DAPI with fluorescence signals) and the exact coordinates of a minimum of 500 nuclei per sample were recorded and evaluated. After the second and third hybridization rounds, slides were evaluated by semi‐automated re‐localization of previously recorded cells, overall allowing the assessment of the ploidy status of the eight chromosomes at a single‐cell level. Cells with no hybridization signals in one or more channels were excluded from further study. Analysis of all nuclei was performed using strict criteria relating to intensity, size, and distribution of FISH signals, as previously described by our group [28]. Only nuclei with at least one chromosome gain (hyperdiploid blasts) were used for further analysis. A minimum of 200 hyperdiploid nuclei with informative data for all hybridization rounds were analyzed. Cut‐off levels for FISH signal positivity were determined by Binomial distribution in the three diploid control samples described above using graphpad prism (San Diego, CA, USA). In patient samples, aneuploidy combinations were considered relevant if each aneusomy level proved to be beyond the assigned cut‐off value.

### 2.4 Single‐cell computational analyses

Shannon entropy indices were obtained for each DX HHD‐B‐ALL sample to assess the differences between CR and REL patients using the formula $H\left(P_{1}\ldots P_{n}\right)=\sum_{i=1}^{n}P_{i}\log_{2}P_{i}$, where *P*_*i*_ is the probability of value and *n* is the number of possible values. The number of gains for each of the eight chromosomes analyzed were used as subclone identifiers (Subclone ID) for each individual cell. Entropy values were calculated for each DX sample using subclone formulas, as previously reported [29]. Hierarchical clustering of both cell clones and individual chromosomes was applied to infer the ordering of chromosomal gains throughout leukemia evolution, based on the frequency of specific chromosomal gains. The Euclidean method was used to compute distance matrices and the agglomeration method was applied to assess complete linkage [30].

For modeling the chromosomal gains in DX samples from CR and REL HHD‐B‐ALL patients, a Random Forest decision tree‐based approach was used to model the relationship of chromosome gains and response to therapy [31]. Gini importance was used to assess which chromosomes were the most relevant for predicting response to therapy [32]. Gini importance measures feature the importance across all the sub‐trees that the Random Forest algorithm generates on a leave‐one‐out cross‐validation strategy (for each sample, we used all the remaining samples for training and the selected instance as test).

### 2.5 Stress tests for prognostic predictors

We performed a stress test for each of the three models derived by Random Forest analysis. Random deviates from a uniform distribution between 0 and each noise level are randomly added or subtracted from the original input data 100 times and predicted again their CR or REL condition. The real conditions were compared with the predictions on the noisy data. The robustness of each model was evaluated by assessing the average number of patients correctly or erroneously predicted along the 100 repetitions and after increasing noise levels.

### 2.6 Validation analyses

The prognostic predictor value obtained by computational modeling of seq‐iFISH data was validated in a blind analysis using an independent cohort of 50 BM samples obtained at disease presentation from the corresponding number of patients with HHD‐B‐ALL. Validation analysis was performed by three‐color FISH using CEP10 (*Spectrum Orange*), CEP18 (*Spectrum Aqua*), and the LSI21 (*Spectrum Green*) probes, following standard FISH protocols. Nuclei with no gains for any probed chromosome were considered normal/healthy hematopoietic cells. A minimum of 200 hyperdiploid nuclei were analyzed per sample.

### 2.7 Statistical analyses

R‐statistics version 4.0.0 (R Foundation for Statistical Computing, Vienna, Austria) was used to perform all single‐cell computational analyses, stress tests and multivariate analyses. Patients that remained disease‐free after a minimum of 5‐year follow‐up were compared with those that relapsed within this timeframe. For analysis of clonal evolution, DX‐REL patient‐matched longitudinal HHD‐ALL samples were compared. *P* < 0.05 was considered statistically significant. Relapse‐free survival (RFS) was calculated from the date of DX to the date of either REL or death, and was estimated with the Kaplan–Meier method and compared with the log‐rank test. Univariate and multivariate analyses using specific chromosomal gains and clonal heterogeneity together with other clinically relevant variables including MRD status after induction treatment, treatment protocol, gender, age and white blood cell count (WBC) at presentation, were performed using the Cox model [33].

## 3 Results

### 3.1 High degree of clonal heterogeneity in HHD‐B‐ALL at DX

Retrospective DX samples from patients with HHD‐B‐ALL were sub‐grouped according to their clinical outcome as (a) patients (*n* = 10) who remained disease‐free without relapse after > 5‐year follow‐up, or (b) patients (*n* = 12) who relapsed (REL) within this timeframe (average time to relapse of 3.5 years; range: 2–7 years). Seven out of these 12 patients (60%) died after the first or second relapse (Fig. 1A and Table 1). No differences in gender distribution, age and WBC were observed between CR and REL HHD‐ALL patients (Fig. 1B,C and Table 1).

To assess clonal heterogeneity in the HHD‐B‐ALL DX samples, we performed seq‐iFISH for single‐cell analysis of the eight chromosomes typically gained in HHD‐B‐ALL (4, 6, 10, 14, 17, 18, 21, and X, Fig. 2A,B). To first establish the cut‐off levels for chromosome gains and losses, we optimized the seq‐iFISH analysis on euploid CD34^+^ HSPCs (*n* = 2) and PBMNCs, which showed consistent rates of chromosome gains < 0.37% and chromosome losses < 5% (Fig. 2C–E and Table S2). As expected, diploid clones were observed in ~ 90% of the cells analyzed (range 88–90.6%) with only minor (range: 0.38–5.2%) subclones showing mainly ‘false’ chromosomal losses (Fig. 2E,F). Remarkably, the order of hybridization steps and the ploidy status of the analyzed chromosomes did not affect the read‐out accuracy of our experimental design (Fig. S1). These results validate the high reliability of our seq‐iFISH analysis of clonal composition in HHD‐B‐ALL. Seq‐iFISH analysis of the HHD‐B‐ALL samples at DX showed high levels of clonal heterogeneity in relation to chromosome copy‐number alterations, as previously observed [25], with major subclones representing between 2.5% and 30.3% of cells (Fig. 2G and Table 2). Notably, whereas the major subclone observed by seq‐iFISH corresponded to that observed by conventional karyotyping analysis in 7 of 22 HHD‐B‐ALL cases (32%), it was different in most cases (15/22, 68%; Table 2). These results revealed high clonal ‘chromosomal’ heterogeneity in HHD‐B‐ALL DX samples.

**Table 2:** Major subclones identified by conventional cytogenetics and seq‐iFISH analyses in HHD‐B‐ALL patients. FISH subclones depict the number of gains observed for chromosomes X, 4, 6, 10, 14, 17, 18, and 21, respectively. Chromosomes identified by seq‐iFISH are labeled in bold. Cases with karyotypes representing the major clone observed by Seq‐iFISH were labeled in blue.

HHD‐B‐ALL	Karyotype	Seq‐iFISH	
		Major clone	%
CR01	58,XY,+**X**,−Y,+**4**,+**6**,+8,+**10**,+11,+12,+**14**,+**17**,+**18**,+**21**,+**21**,+22 (SNP6)	01110111	4.74
CR02	53,XX,+**X**,+**X**,+**6**,+**14**,+**17**,+**21**,+**21** (SNP6)	10001102	7.95
CR03	55,XXYY,+**X**,+Y,+**4**,+**6**,+12,+15,+**18**,+**21**	11111111	7.12
CR04	54,XX,+**X**,+**6**,+8,+**14**,+**17**,+**18**,+**21**,+**21**[15] / 54,idem,−13,+mar[11]/46,XX[19]	10101102	7.39
CR05	57,XX,+**X**,+**X**,+**4**,+**6**,+8,+**10**,+**14**,+**17**,+**18**,+**21**,+**21** (SNP6)	21111111	10.85
CR06	54,X,+**X**,Y,+**6**,+**10**,+**14**,+**17**,+**18**,+**21**,+mar[30]	10111111	12.95 ^a^
CR07	55,XXY,+**X**,+3,+**4**,+**6**,+**10**,+**14**,+**18**,+**21**,+**21**/46,XY	11111012	8 ^a^
CR08	60,XY,+**X**,+**4**,+**6**,+7,+8,+9,+**14**,+**17**,+**18**,+**21**,+**21**,+3mar/46,XY	11112122	3.96
CR09	57,XXY,+**X**,+**4**,+**6**,+8,+**10**,+**10?**,+13,+**14**,+**17**,+**18**,+**21**[14]/46,XY[16]	11121111	2.55 ^a^
CR10	54,XXX,+**X**,+**4**,+**6**,+8,inv(9)(p11q12),+**14**,+**17**,+**18**,+**21**[22]/46,XX,inv(9)(p11q12)[2]	11101111	7.72 ^a^
REL01	59,XY,+?**X**,+**4**,+**6**,+7,+8,+12,+**14**,+?add(17)(p1),+**18**,+**18**,+**21**,+der(?)t(?;5)(?;q?13),+mar[6]/63,idem,dup(1)(q2q3),+22,inc[7]/46,XY[5]	11121121	3.61
REL02	51,XX,+**X**,+8,+**14**,+**21**,+**21**[5]/46,XX[9]	10001002	30.32 ^a^
REL03	54,XY,+**X**,+**4**,+**6**,+**10**,+**14**,+add(19)(p13),+20,+**21**[cp8]	11111102	10.36
REL04	46,XX[20]/High hyperdiploid by FISH	01112002	22.3
REL05	52‐56,XY,+**X**,dup(1)(q25q44),+**4**,+8,+**10**,+11,+**14**,+**18**,+**21**,+**21**,+mar[cp7]/46,XY[3]	11011002	9.09
REL06	46,XY[20]/High hyperdiploid by FISH	11111111	5.83
REL07	56,XY,+**X**,ins(1;?),(q21.3;?),+**4**,+**6**,+**14**,+**17**,+**18**,+**21**,+**21**,+22,+mar[5]/46,XY[5]	11101112	12 ^a^
REL08	High hyperdiploid by FISH	10001002	11.57
REL09	51,XX,+**X**,+**6**,+**14**,+**21**,+**21**[16]/51,idem,add(9)(q34)[2]/46,XX[2]	10101002	10.85 ^a^
REL10	53,XX,+**4**,+**6**,+**14**,+**17**,+**18**,+2mar[8]/46,XX[22]	11101111	7.86
REL11	59,XXY,+**X**,der(1)(q?),+**4**,+5,+**6**,+8,+**10**,+11,+**18**,+**18**,+**21**,+22,mar[9]/46,XY[41]	12120022	8.45
REL12	57,XY,der(1)(p?q?),+**4**,**10mar**[56%]/46,XY[44%]	11121012	10.61

^a^ Major clone identified by seq‐iFISH corresponds with the clone detected by conventional karyotyping.

### 3.2 Differential rates of specific chromosome gains and clonal heterogeneity at DX between CR and REL HHD‐B‐ALL patients

**Fig. 3:**
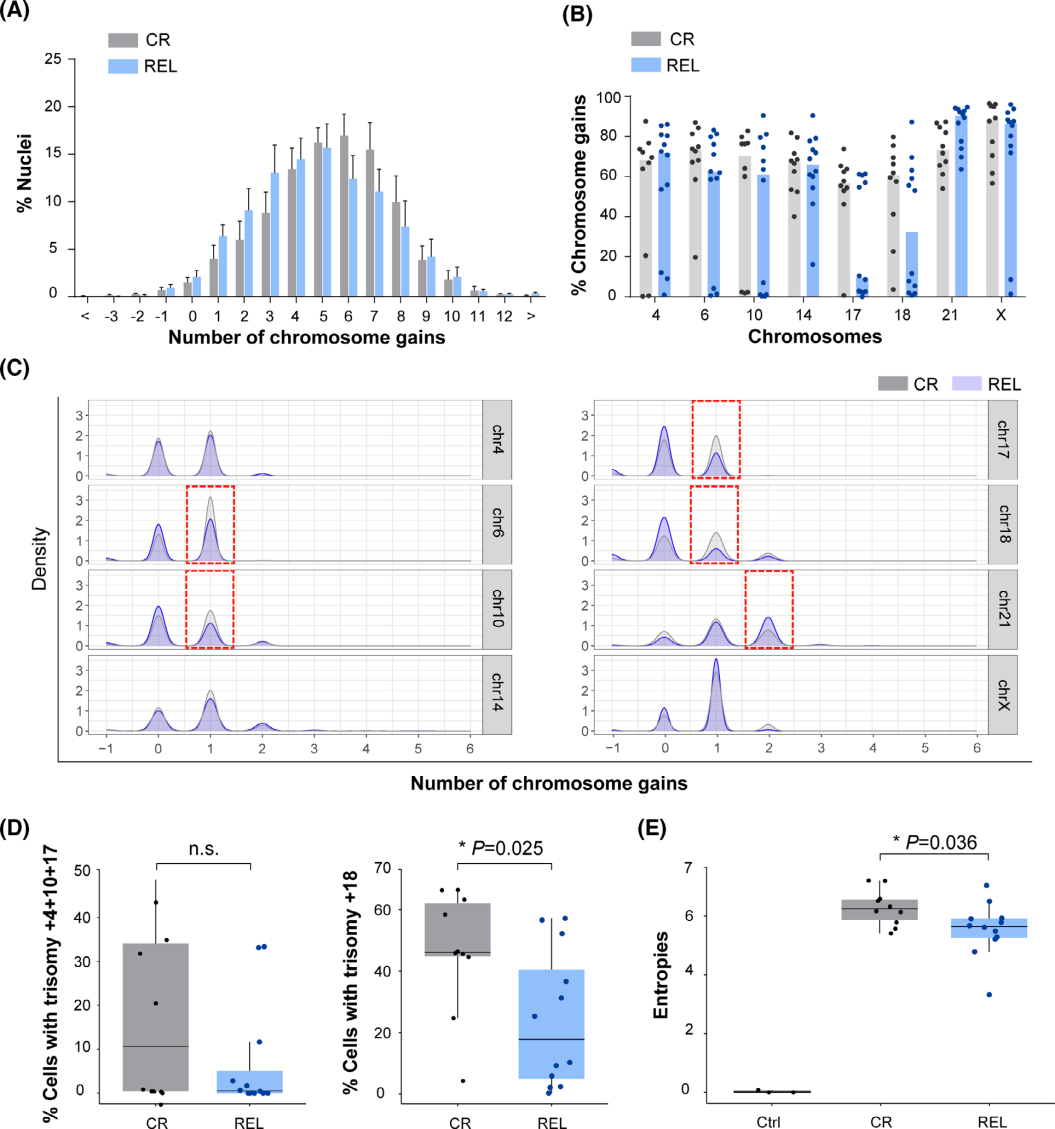
Differential rates of specific chromosome gains and clonal heterogeneity in diagnostic (DX) samples from complete remission (CR) and relapsed (REL) high‐hyperdiploid B‐cell acute lymphoblastic leukemia (HHD‐B‐ALL patients). (A) Number of total chromosomal gains in DX samples from CR and REL HHD‐B‐ALL patients. Graphs represent the mean value and error bars represent the SEM. (B) Frequency of chromosomal gains for the indicated chromosomes in DX samples from CR and REL HHD‐B‐ALL patients. Graphs represent the median values of total gains for each chromosome and dots represent the values obtained for individual patients. (C) Chromosomal gains as observed by density values in single‐cell computational analysis for the indicated chromosomes distinguishing the contribution of trisomies and tetrasomies. Red boxes indicate the differences observed in DX samples from CR and REL HHD‐B‐ALL patients. (D) Box plots comparing the frequency of cells harboring triple trisomies 4, 10, and 17 (*left*) and single trisomy 18 (*right*) between DX samples from CR and REL HHD‐B‐ALL patients. (E) Box plots comparing the clonal heterogeneity as observed by Shannon entropy values of data between controls (ctrl) and DX samples from CR and REL HHD‐B‐ALL patients. Boxes represent the quartiles 25–75 and horizontal lines represent the mean value. Error bars represent the standard deviation (SD); *n* = 3 ctrl, *n* = 10 CR, and *n* = 12 REL HHD B‐ALL patients; two‐sided unpaired *t*‐test. *P‐value < 0.05. [Colour figure can be viewed at wileyonlinelibrary.com]

Seq‐iFISH analysis performed on DX samples revealed no differences in the number of total chromosomal gains observed in CR *versus* REL HHD‐B‐ALL patients, with a peak between four and six chromosomal gains in both groups (Fig. 3A). However, different rates of specific chromosome gains were observed between CR and REL HHD‐B‐ALL patients. Increased rates of gains of chromosomes 17 and 18, and moderate increased rates of gains of chromosomes 6 and 10, were found in DX samples from CR HHD‐B‐ALL patients. In contrast to the increased rates of chromosomal gains in CR HHD‐B‐ALL patients, moderately higher rates of chromosome 21 gains were found in REL HHD‐B‐ALL patients (Fig. 3B,C). These chromosomal gains were mainly trisomies of all chromosomes analyzed, except for chromosome 21, which showed similar trisomy rates between groups and higher tetrasomy rates in REL HHD‐B‐ALL samples (Fig. 3C).

Specific trisomies, such as triple trisomies for chromosomes 4, 10, 17, and single trisomy for chromosome 18 have been reported to have prognostic value in HHD‐ALL based on conventional cytogenetics analysis [14, 17, 34]. We next analyzed the percentage of DX blasts bearing either trisomies 4, 10, 17, or trisomy 18 in our cohort, irrespective of other chromosomal gains. In line with previous studies, we found that the frequency of DX blasts bearing either trisomies 4, 10, 17 (*P* > 0.05), or trisomy +18 (*P* = 0.025) was higher in CR than in REL HHD‐B‐ALL samples (Fig. 3D), suggesting that at least trisomy 18 may have prognostic value for HHD‐B‐ALL. Beyond specific chromosomal gains, the levels of CIN have been associated with tumor progression in different types of cancer [35, 36, 37, 38, 39]. To assess whether CIN levels are associated with HHD‐B‐ALL outcome, we next analyzed the levels of clonal heterogeneity in our samples, which is directly associated with CIN [40], based on the analysis of the entropy values obtained by computational analysis of single‐cell data [29]. We found significantly higher levels of clonal heterogeneity in DX samples from CR than from REL HHD‐B‐ALL patients, as revealed by increased entropy values (Fig. 3E), suggesting that clonal heterogeneity may also represent a favorable prognostic factor in HHD‐B‐ALL at DX. Collectively, our data show that HHD‐B‐ALL patients show a high and variable degree of clonal heterogeneity with differential rates of specific chromosomal gains between CR and REL HHD‐B‐ALL patients. Notably, the higher levels of clonal heterogeneity observed in DX samples from CR HHD‐B‐ALL suggest its potential as a biomarker for HHD‐B‐ALL outcome, suggesting that one of the subclones present at DX emerges as the dominant clone at REL.

### 3.3 HHD‐B‐ALL shows hierarchical chromosome gains without specific clones associated with REL

**Fig. 4:**
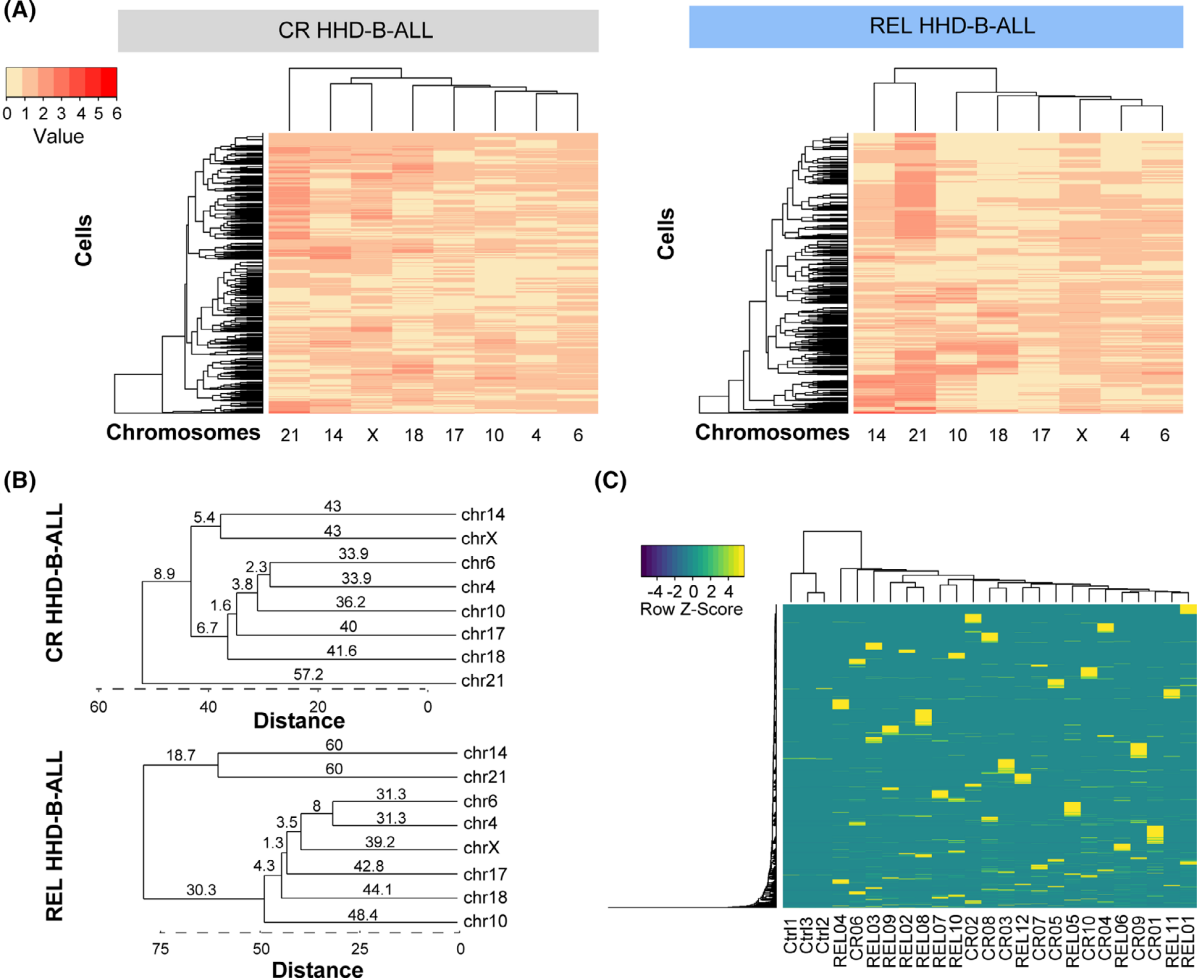
High‐hyperdiploid B‐cell acute lymphoblastic leukemia (HHD‐B‐ALL) shows hierarchical chromosome gains without specific clones associated with relapse. (A) Heatmaps depicting the acquisition of chromosomal gains in all the cells analyzed in diagnostic (DX) samples from complete remission (CR; *left; n* = 2983 cells) and relapse (REL; *right*; *n* = 3142 cells) HHD‐B‐ALL patients who had achieved minimal residual disease (MRD)‐negativity post‐induction therapy. Lines represent individual clones and columns represent individual chromosomes. The number (value) of chromosomal gains per clone is represented as different red color intensities. (B) Summarized dendrograms using data from (A). Euclidean distances are shown for each cluster identified. (C) Heatmap depicting the unique subclones in control (Ctrl), CR and REL HHD‐B‐ALL samples. The color code represents the row *Z*‐score for individual clones. [Colour figure can be viewed at wileyonlinelibrary.com]

A sequential and ordered acquisition of chromosomal gains has been reported in HHD‐B‐ALL [25, 41]. To study the potential order of chromosomal gains in DX samples from both CR and REL HHD‐B‐ALL patients, we next performed a hierarchical cluster analysis of the acquisition of chromosomes using the number of overall gains in all the cells analyzed for each group at DX. Such hierarchical cluster analysis revealed specific associations regarding chromosome gains that did not substantially differ between CR and REL HHD‐B‐ALL patients (Fig. 4A). Three major clusters were distinguished in DX samples from CR HHD‐B‐ALL patients at a Euclidean distance of 48.3. Chromosome 21 lies at the base of the tree, followed by chromosomes 14 and X that cluster separately from the remaining chromosomes which, in turn, also form smaller sub‐clusters (Fig. 4A,B). Two major clusters were observed in DX samples from REL HHD‐B‐ALL patients with chromosomes 21 and 14 at the base of the tree, separated from the remaining chromosomes by a Euclidean distance of 11.6 (Fig. 4A,B). Hierarchical cluster analyses in individual samples showed variability between individuals but with a marked tendency for chromosomes 21, 14 or X to cluster together at the base of the tree (Figs S2 and S3). In addition, chromosomes 4 and 6 clustered together as a unique sub‐cluster separated at higher Euclidean distances from the others, suggesting that these chromosomes were gained later during leukemogenesis both in CR and REL HHD‐B‐ALL. Our results confirm a hierarchical ordering in chromosomal gains in HHD‐B‐ALL, with chromosomes 21, 14, and X gained earlier and chromosomes 4 and 6 later during leukemogenesis.

To assess whether the hierarchical order of chromosomal gains results in the formation of specific clones that can be associated with REL in HHD‐B‐ALL, we next performed a hierarchical cluster analysis of those unique subclones observed at DX in each analyzed sample. Results highlighted an extensive clonal heterogeneity in HHD‐B‐ALL DX samples with no specific clones (clustering) associated with REL (Fig. 4C).

### 3.4 Computational modeling identifies trisomies 18 and 10 as a relapse predictor in MRD‐negative HHD‐B‐ALL

**Fig. 5:**
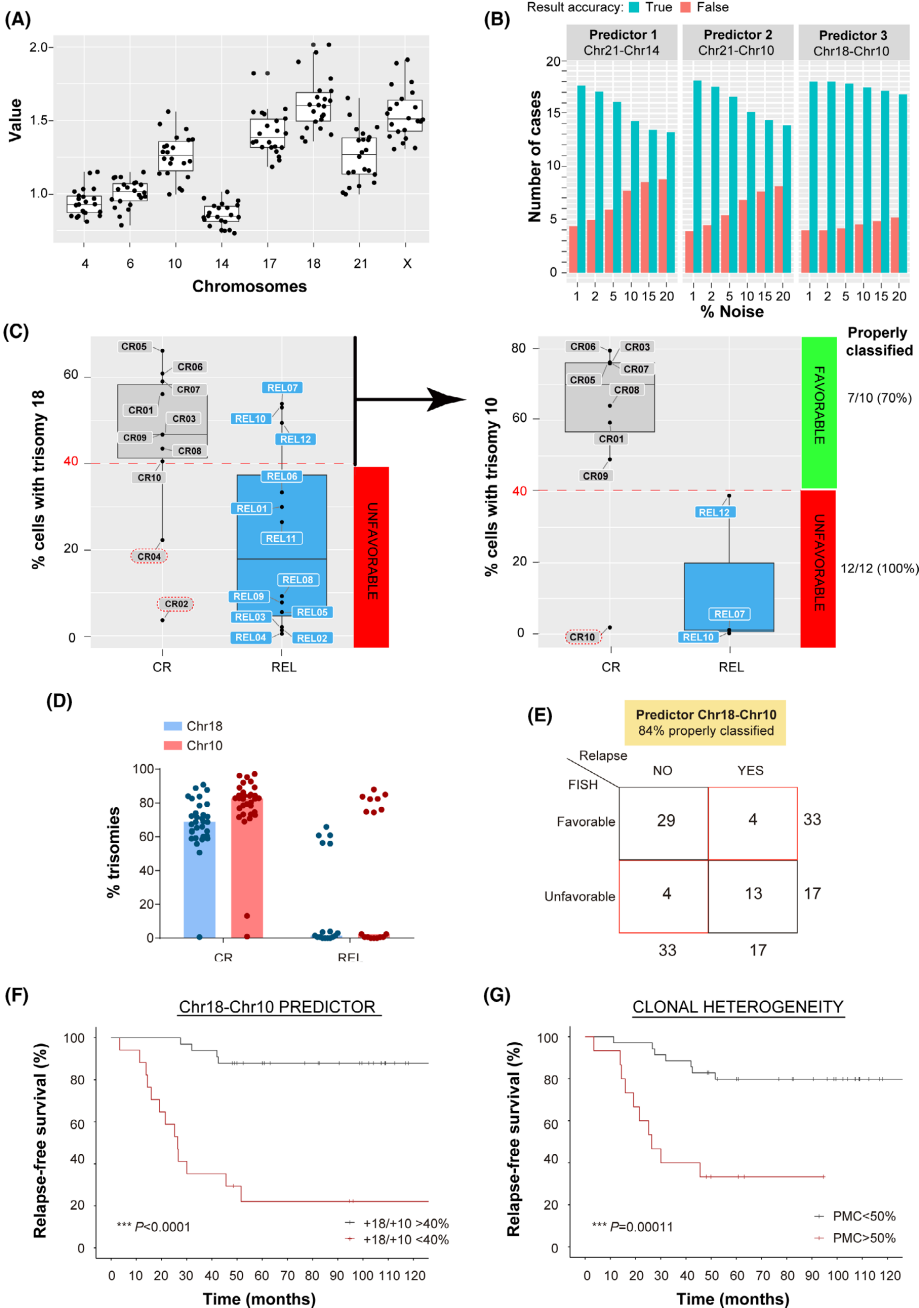
Trisomies 18 and 10 and clonal heterogeneity are robust relapse risk predictors in high‐hyperdiploid B‐cell acute lymphoblastic leukemia (HHD‐B‐ALL) who had achieved minimal residual disease (MRD)‐negativity post‐induction therapy. (A) Contribution of the indicated trisomies at predicting clinical outcome; complete remission (CR) versus relapse (REL). Dots represent individual patient Gini importance values. Boxes represent the quartiles 25–75 and horizontal lines represent the mean value. Error bars represent the SD; *n* = 10 CR and 12 REL patient samples. (B) Stress tests on the indicated predictors for risk classification at diagnosis (DX) of HHD‐B‐ALL patients (*n* = 22). Blue bars represent the number of patients properly classified according to disease outcome (CR versus REL) and red bars the number of patients erroneously classified. (C) Classification based on DX samples of HHD‐B‐ALL patients according to risk predictor 3 as either CR (favorable, % trisomies 18 and 10 > 40%) or REL (unfavorable, % trisomies 18 and 10 < 40%) risk groups. Number and percentage of properly classified patients are indicated in the right. (D) Frequency of the indicated chromosome gains in an independent and blind validation cohort of DX samples of favorable (CR, *n* = 33) and unfavorable (REL, *n* = 17) HHD‐B‐ALL patients by iFISH analysis of chromosomes 18 and 10. Graphs represent the median values and dots represent the values obtained for individual patients; *n* = 200 nuclei per sample. (E) Classification of HHD‐B‐ALL patients as CR or REL using the chr18‐chr10 risk predictor (predictor 3) after blind FISH analyses. The DX samples from the validation cohort were initially blind‐grouped as favorable or unfavorable risk based on chr18–chr10 risk predictor results, and then correlated with relapse information (No versus Yes) available from the clinic. 96% (48/50) of patients had achieved MRD negativity after induction therapy. The total number of patients for each group is indicated outside the quadrant. Note that 84% of the 50 HHD‐B‐ALL patients used in this blind and independent validation cohort were properly classified using predictor 3. (F,G) Kaplan–Meier curves for relapse‐free survival (RFS) of HHD‐B‐ALL patients grouped according to the chr18–chr10 risk predictor (F) and clonal heterogeneity defined by the percentage of major clone (PMC) (G) after blind FISH analyses; *n* = 50 HHD‐B‐ALL samples collected at diagnosis (DX). Analyses for panels (F and G) were performed with the hazard ratios obtained with cox multivariate analyses. [Colour figure can be viewed at wileyonlinelibrary.com]

We next examined whether specific combinations of chromosomal gains in DX samples could distinguish MRD‐negative HHD‐B‐ALL patients with favorable (CR) versus unfavorable (REL) clinical outcome. We employed a machine learning approach, which ranks the relative contribution of each chromosome analyzed in distinguishing between CR and REL HHD‐B‐ALL [32]. This analysis highlighted trisomies 18, X, 17, 21, and 10 as potential informative parameters classifying samples as CR or REL (Fig. 5A). Using these data, we next used decision trees to test which combinations of these five chromosomal gains best classified HHD‐B‐ALL patients. Among all possible permutations, only three displayed significant predictor potential: chromosomes 21 and 14, chromosomes 21 and 10, and chromosomes 18 and 10 (Fig. 5B). The reliability of these combinations of chromosomal gains found in DX samples in predicting favorable or unfavorable clinical evolution of the HHD‐B‐ALL patients was confirmed by ‘stressing’ the data through a gradual increase in the percentage of noise [31, 32]. Stress tests allowed assessment of the robustness of each combination of chromosomal gains in relation to biological and technical variability. These analyses revealed that the predictor‐3, based on trisomies 18 and 10, was the most stable risk predictor associated with a lower rate of misclassification events, a stable reproducibility, even at high noise levels, and with an accuracy of 82% (18 of 22 cases were correctly classified; Fig. 5B). We thus established a risk predictor for HHD‐B‐ALL patients based on a two‐step classification using chromosomes 18 and 10 trisomy rates. Patients were first classified by the percentage of chromosome 18 gains, with a threshold of 40%. Cases below this threshold were accurately classified as unfavorable‐risk (REL) patients. Next, cases above this threshold were further refined based on the percentage of chromosome 10 gains, with a threshold of 40%. Those HHD‐B‐ALL patients below this second threshold were also classified as unfavorable risk and cases above were regarded as favorable prognosis (Fig. 5C). This computational modeling‐based risk predictor correctly classified in DX samples HHD‐B‐ALL patients with favorable and unfavorable outcome with a 70% and 100% accuracy, respectively.

### 3.5 An independent cohort of DX samples validates the high reliability of low levels of trisomy +18+10 and low levels of clonal heterogeneity as relapse predictors in MRD‐negative HHD‐B‐ALL

To further validate our computationally defined risk predictor in relation to clinical outcome, we performed a blind validation test using a larger independent cohort of HHD‐B‐ALL DX samples from 50 patients (48/50 (96%) MRD‐negative after induction therapy) selected to include both favorable (remain disease‐free after 5 years) and unfavorable (relapsed within 5 years) cases (66% versus 34%, respectively; Table S1). For this validation, we analyzed the aneuploidy rates for chromosomes 18, 10, and 21 by three‐color iFISH. Chromosome 21 was used as a control to discriminate hyperdiploid leukemic cells from normal hematopoietic cells disomic for chromosomes 18 and 10. Consistent with previous data from seq‐iFISH, these validation results showed significantly higher frequencies of both chromosome 18 and 10 trisomies in DX samples from those HHD‐B‐ALL patients who remained disease‐free as compared with those that relapsed after treatment (Fig. 5D). Remarkably, HHD‐B‐ALL patient classification at DX using the trisomies 18‐ and 10‐based risk predictor was highly reliable, with 42/50 (84%) of the patients correctly classified as either disease‐free or relapsed (Fig. 5E and Table S1).

To evaluate the prognostic value of this computationally defined risk predictor in the outcome of HHD‐B‐ALL patients (48/50 (96%) MRD‐negative after induction therapy), we next performed univariate and multivariate statistical analyses of different clinically relevant parameters, including age, gender, WBCs, MRD status, treatment protocol and the trisomies 18‐ and 10‐based risk predictor using a Cox regression model. Of note, both univariate and multivariate analyses showed that the relapse predictor based on low levels (cut‐off, 40%) of trisomies 18 and 10 was the only independent risk factor associated with higher REL rates (hazard ratio = 11.1; 95% confidence interval = 3.55–34.8, *P* < 0.001; Table 3). In fact, within our validation cohort, statistically significant higher relapse‐free survival (RFS) rates were observed in patients with a favorable (trisomies +18+10 > 40%) than in patients with unfavorable (trisomies +18+10 < 40%) risk predictor, with a 10‐year RFS of 87.9% and 22.1%, respectively (*P* < 0.0001; Fig. 5F).

**Table 3:** Evaluation of the FISH risk predictor as a prognostic factor in HHD‐B‐ALL. Univariate and multivariate analyses comparing different clinical parameters and the chromosome 18–10 trisomies‐based risk predictor prognostic value in a blind validation of an independent HHD‐B‐ALL cohort (*n* = 50 patients). CI, 95% confidence interval; MRD, MRD‐positive after induction; WBC, white blood cell count.

Variable	Univariate			Multivariate		
	Hazard ratio	CI	*P‐*val	Hazard ratio	CI	*P‐*val
Age^a^	1.47	0.52–4.2	0.47	1.16	0.48–3.98	0.5
Sex (male)	2.85	0.65–12.5	0.2	2.04	0.45–9.29	0.4
WBC	1.01	0.99–1.01	0.9	1	0.99–1.02	0.8
MRD	3.54	0.80–15.64	0.09	4.45	0.36–55.1	0.2
Unfavorable FISH risk predictor	11.9	3.82–36.9	< 0.001	11.1	3.55–34.8	< 0.001
Treat (PETHEMA)	*Reference*			*Reference*		
Treat (SHOP)	0.74	0.15–3.67	0.7	1.73	0.24–12.3	0.6
Treat (AIEOP‐BFM)	2.14	0.74–6.19	0.2	1.57	0.47–5.23	0.5
Treat (UKALL2003)	10.2	1.11–93.54	0.04	0.87	0.04–20.1	0.9

^a^ Per 5‐year increase.

Using seq‐iFISH and computational modeling we have established that HHD‐B‐ALL DX samples with smaller relative representation (percentage) of the major clone (PMC) display higher entropy, indicative of higher clonal heterogeneity (Table 2 and Fig. 3E). The levels of clonal heterogeneity analyzed in DX samples were, in fact, higher in CR than REL HHD‐B‐ALL patients, suggesting that the levels of clonal heterogeneity may also predict the clinical evolution of HHD‐B‐ALL patients. To assess the prognostic value of clonal heterogeneity, we used the PMC obtained in our three‐color iFISH analyses. To obtain a cut‐off value for the PMC, we first dichotomized this variable using maximally‐selected rank statistics, finding that the best threshold for PMC was 50%. Our results revealed, within our cohort, a significantly higher RFS in patients with PMC ≤ 50% (RFS beyond 5 years: 79.8% versus 33.3%, *P* = 0.0001, Fig. 5G), thus demonstrating that lower levels of clonal heterogeneity represent a risk factor associated with REL HHD‐B‐ALL. Importantly, the rates of trisomies 18 and 10 were associated with the PMC, as revealed by the negative correlation between both variables for each patient (*P* < 0.0001, Fig. S4), suggesting that the levels of aneuploidy correlate with CIN in HHD‐B‐ALL. These results validate our computationally defined risk predictors in HHD‐B‐ALL, and demonstrate that low rates of trisomies 18 and 10, and low levels of clonal heterogeneity are risk factors in HHD‐B‐ALL.

### 3.6 Longitudinal analysis of matched DX‐REL HHD‐B‐ALL samples reveals distinct patterns of clonal evolution at REL

**Fig. 6:**
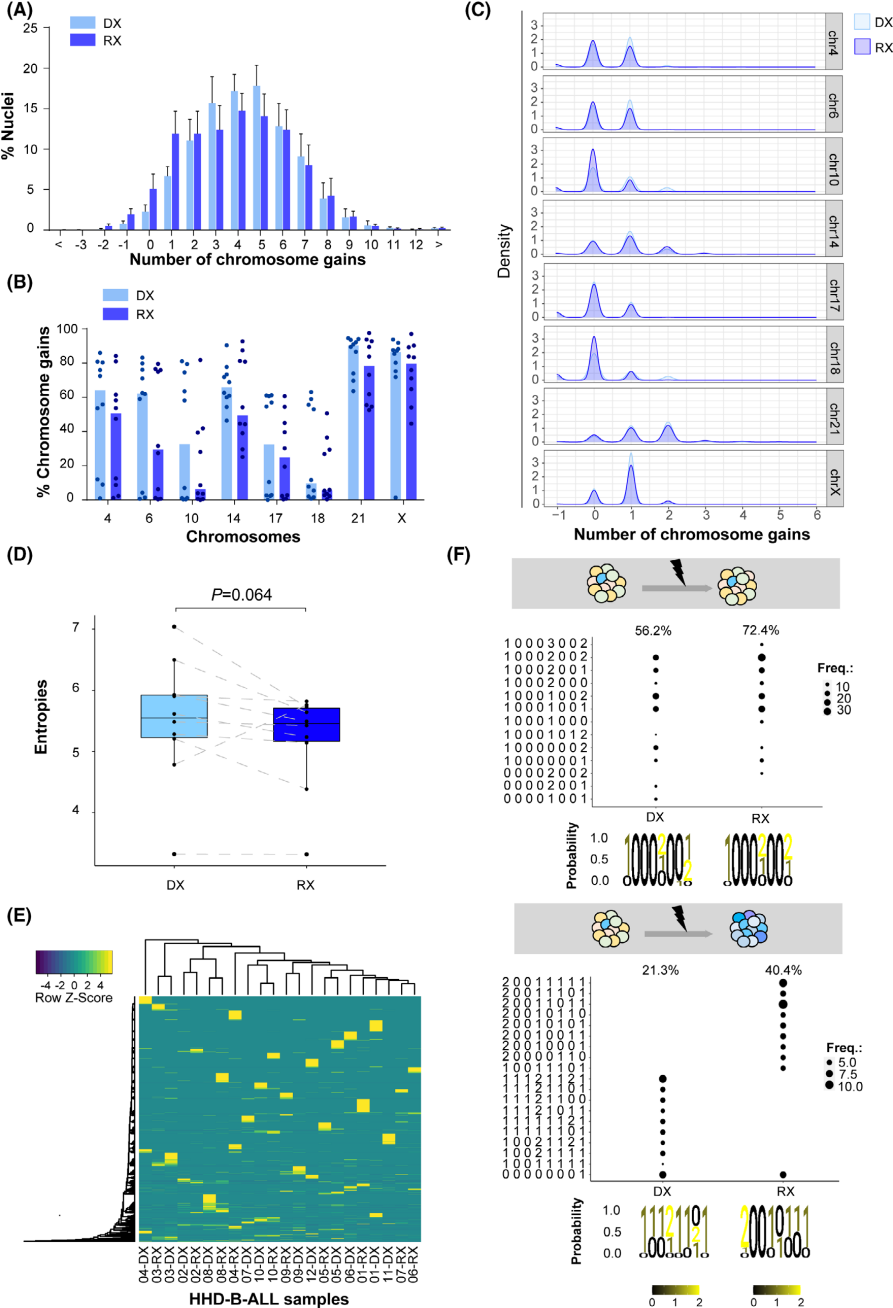
Longitudinal analysis of matched diagnostic‐relapse high‐hyperdiploid B‐cell acute lymphoblastic leukemia (HHD‐B‐ALL) samples reveals distinct patterns of clonal evolution at REL. (A) Number of total chromosomal gains in matched diagnostic (DX) and relapse (REL) HHD‐B‐ALL samples. Graphs represent the mean value and error bars represent the SEM; *n* = 20 samples (10 DX and 10 REL). (B) Frequency of chromosomal gains for the indicated chromosomes in matched DX and REL HHD‐B‐ALL samples. Graphs represent the median values and dots represent the values obtained for each single sample. (C) Number of chromosomal gains in matched DX and REL HHD‐B‐ALL samples as observed by density values in single‐cell computational analysis for the indicated chromosomes. (D) Box plot comparing the clonal heterogeneity as observed by Shannon entropy values between matched DX and REL HHD‐B‐ALL samples. Boxes represent the quartiles 25—75 and horizontal lines represent the mean value. Error bars represent the SD; two‐sided paired *t*‐test. (E) Heatmap depicting the unique subclones in matched DX and REL HHD‐B‐ALL samples. The color code represents the row *Z*‐score for individual clones. On the top, dendrogram with hierarchical cluster analyses of samples regarding clonal composition similarity. (F) Single‐cell analysis of the major clones observed in matched DX and REL HHD‐B‐ALL samples. Representative analyses of the two different patterns of chromosomal clonal evolution observed, with the major leukemic clones being either shared (*top*) or distinct (*bottom*) in matched DX and REL samples. The subclone formulas on the left indicate the number of gains for chromosomes X, 4, 6, 10, 14, 17, 18, and 21, respectively. The size of the black circles represents the proportion of cells showing that indicated subclone in each sample. [Colour figure can be viewed at wileyonlinelibrary.com]

We next sought to analyze the clonal evolution during disease progression through longitudinal analyses of available matched DX‐REL samples from 10 HHD‐B‐ALL patients (Table 1). Results showed no major difference in the number of total chromosomal gains between matched DX and REL samples, peaking between four and five chromosomal gains in both groups (Fig. 6A). The rates of individual chromosomal gains were consistently reduced for all chromosomes in the REL samples compared with the matched DX samples (Fig. 6B,C). Analysis of clonal heterogeneity in matched DX‐REL samples showed a trend towards reduced entropy levels in REL samples (*P* = 0.064), with six of 10 patients showing lower entropy levels in the REL samples (Fig. 6D), supporting previous data on DX samples of high levels of clonal heterogeneity as a favorable prognostic factor in HHD‐B‐ALL.

To study the clonal evolution in HHD‐B‐ALL during disease progression, we next performed a hierarchical cluster analysis of those unique subclones observed in matched DX‐REL samples and found that in 7/10 (70%) of the HHD‐B‐ALL patients the DX‐REL samples clustered together (Fig. 6E). Interestingly, analyses of clonal composition in matched DX‐REL samples revealed two different patterns of chromosomal clonal evolution: (a) a pattern where the major leukemic clones are shared in DX and REL (7/10 of the patients), and (b) a pattern with a large clonal replacement from DX to REL (3/10 of the patients; Fig. 6F and Fig. S5). Collectively, our longitudinal analysis of matched DX‐REL HHD‐B‐ALL samples reveals distinct patterns of clonal evolution at REL.

## 4 Discussion

In this study, we took advantage of primary DX and REL BM samples from a large cohort of patients with HHD‐B‐ALL with either favorable or unfavorable clinical outcome (after MRD negativity post‐induction treatment), in order to comprehensively characterize clonal heterogeneity, specific chromosomal gains and clonal evolution. An important goal was to assess the potential prognostic impact of specific chromosomal gains and clonal heterogeneity in HHD‐B‐ALL. This study is technically sound because seq‐iFISH analysis was coupled with single‐cell computational modeling allowing for a comprehensive investigation of the eight chromosomes typically gained in HHD‐B‐ALL at a single‐cell level.

Our data showed a high degree of clonal ‘chromosomal’ heterogeneity in HHD‐B‐ALL patients at DX, with major subclones commonly representing < 30% of the leukemic cells. The major subclones identified by seq‐iFISH usually differed from those observed using conventional karyotyping. Earlier studies have reported contradictory data on the presence of clonal heterogeneity in HHD‐B‐ALL which depend on the methodology of analysis used [9, 23, 25, 41]. Consistent with our data, clonal heterogeneity was observed by iFISH [23, 24, 25], spectral‐karyotyping [42], single‐cell next‐generation sequencing [43] and by cell division studies directly assessing chromosome segregation in primary leukemic blasts [21]. Our results reinforce that conventional karyotyping fails to reflect the actual clonal heterogeneity in HHD‐B‐ALL patients, suggesting that clonal selection seen in conventional karyotyping analysis likely arises from the cell culture needed for karyotyping, which may mask the actual clonal composition of HHD‐B‐ALL samples.

Importantly, the clonal heterogeneity revealed by our seq‐iFISH analysis suggested the presence of CIN in HHD‐B‐ALL. CIN has emerged as a prognostic factor in different types of cancer, typically being associated with unfavorable clinical outcomes [44, 45]. However, there is an extensive body of evidence suggesting that high CIN levels can also be found associated with favorable outcomes due to a reduced tumor‐cell viability [35, 46]. Indeed, increasing CIN levels represent a strategy to selectively target cancer cells [47]. To assess whether CIN levels are associated with HHD‐B‐ALL outcome, we investigated the levels of clonal heterogeneity based on analysis of the entropy values obtained by computational analysis of single‐cell data [29]. Our results show variable levels of clonal heterogeneity in DX samples of HHD‐B‐ALL patients, with significantly higher levels in DX samples from HHD‐B‐ALL patients showing a favorable clinical outcome. To assess the prognostic value of clonal heterogeneity, we used the PMC and confirmed in the validation cohort a significantly higher RFS in patients with PMC ≤ 50%, thus demonstrating that lower levels of clonal heterogeneity represent a risk factor associated with unfavorable outcome.

Contradictory data exist regarding the predictive power of different chromosomal abnormalities on HHD‐B‐ALL outcome [14, 16, 17, 18, 34]. Our results revealed that the rates of specific chromosomal gains, similar to the levels of clonal heterogeneity, differ between HHD‐B‐ALL patients with favorable or unfavorable clinical outcomes. Our results are in line with previous data suggesting trisomy 18 as a favorable prognostic factor in HHD‐B‐ALL [17], as rates of trisomy 18 in DX samples were significantly higher in CR than in REL patients. Moreover, computational modeling of seq‐iFISH single‐cell data allowed us to define a risk predictor based on the rates of trisomies 18 and 10. This computationally defined risk predictor was validated in an independent cohort of HHD‐B‐ALL patients and correctly classified 84% of patients according to clinical outcome, confirming the high reliability of the trisomies 18 and 10 risk predictor. In fact, and similar to clonal heterogeneity, univariate and multivariate analyses revealed high levels (> 40%) of trisomies 18 and 10 as an independent favorable prognostic factor in childhood HHD‐B‐ALL with a 10‐year RFS of 88% versus 22%. Importantly, the rates of trisomies 18 and 10 were inversely correlated with the PMC, which further suggested that the levels of aneuploidy correlate with CIN in HHD‐B‐ALL. Of note, almost all (96%) patients in the validation cohort had reached MRD negativity after induction treatment, thus highlighting the reliability of both risk predictors in stratifying those HHD‐B‐ALL patients who, even having achieved MRD negativity are at risk of progression/relapse. Furthermore, the multivariate analysis confirmed the power of both risk predictors in stratifying regardless of the treatment protocol.

Single‐cell computational modeling also provided information about clonal evolution in HHD‐B‐ALL samples. On one hand, and consistent with previous data [25], our analysis revealed a high‐order hierarchy in relation to chromosomal gains in HHD‐B‐ALL, with specific chromosomes being gained together at different stages of disease initiation/evolution. A hierarchical cluster analysis of those unique subclones observed at DX in each analyzed sample highlighted an extensive clonal heterogeneity in HHD‐B‐ALL DX samples with no specific clones associated with relapse, suggesting that specific chromosomal gains rather than specific clones were associated with disease outcome in HHD‐B‐ALL. On the other hand, longitudinal analyses using paired DX‐REL HHD‐B‐ALL samples revealed two different patterns of chromosomal clonal composition at relapse: (a) major leukemic clones shared in DX and REL, and (b) large clonal replacement from DX to REL. This latter clonal evolution pattern may reflect the emergence of leukemic subclones resistant/adapted to chemotherapy‐induced clonal pressure. Although the number of matched DX‐REL samples is relatively limited to reach definitive conclusions, it is noteworthy that the patients with such clonal replacement from DX to REL had a worse outcome and they all succumbed to the disease.

HHD‐B‐ALL is one of the most common malignancies in children [12] and up to 20% of these patients eventually relapse. In fact, in absolute numbers there are more cases of relapsed HHD‐B‐ALL than *de novo* diagnoses of other unfavorable molecular‐subtypes of B‐ALL [48]. Therefore, an improved risk‐stratification of HHD‐B‐ALL patients is crucial to prospectively identify those patients, who, having achieved MRD negativity after induction treatment, remain at high risk of progression/relapse.

## 5 Conclusions

Here, we report the levels of clonal heterogeneity and the rates of trisomies 18 and 10 as robust and independent relapse risk predictors in HHD‐B‐ALL. FISH analyses are commonly used in hemato‐oncology clinical laboratories for B‐ALL diagnosis, as a complement for refining diagnosis after karyotyping or in cases with failed cytogenetics [49]. Our results provide a panel of chromosomes for application of routine FISH testing of HHD‐B‐ALL and offer a reliable prognostic sub‐stratification of HHD‐B‐ALL patients at DX.

## Conflict of interest

The authors declare no conflict of interest.

## Author contributions

MR‐M, JLT, VCR‐C, AB, and BL‐M performed the experiments. CS, MO, PV, MLB, JN, MR‐O, AM, JLF, EC, MC, PB, GE, JB, MB, JMH‐R, MJC, GC, and CJH: provided clinical samples and biological data. JB and CB: designed experiments and interpreted data. PM: conceived the study, designed experiments, interpreted the data, financially supported the study and wrote the manuscript. OM: conceived the study, designed and performed experiments, analyzed/interpreted the data, and wrote the manuscript.

### Peer review

The peer review history for this article is available at https://publons.com/publon/10.1002/1878-0261.13276.

## Supporting information


**Fig. S1.** (Related to Fig. 2). Read‐out accuracy and reliability of Seq‐iFISH analyses.
**Fig. S2.** (Related to Fig. 4). Hierarchical chromosomal gains in the indicated complete remission (CR) high‐hyperdiploid B‐cell acute lymphoblastic leukemia (HHD‐B‐ALL) patients.
**Fig. S3.** (Related to Fig. 4). Hierarchical chromosomal gains in the indicated relapsed (REL) high‐hyperdiploid B‐cell acute lymphoblastic leukemia (HHD‐B‐ALL) patients.
**Fig. S4.** (Related to Fig. 5). Aneuploidy levels are associated with chromosome instability (CIN).
**Fig. S5.** (Related to Fig. 6). Individual longitudinal analysis of matched diagnostic‐relapse (DX‐REL) high‐hyperdiploid B‐cell acute lymphoblastic leukemia (HHD‐B‐ALL).

**Table S1.** Cytogenetic and clinical data of all the childhood high‐hyperdiploid B‐cell acute lymphoblastic leukemia (HHD‐B‐ALL) samples used for blind validation analysis.

**Table S2.** Levels of ‘false’ gains and losses observed by Seq‐iFISH analysis in the indicated control (Ctrl) samples.

## Data Availability

The datasets used and/or analyzed during the current study are available from the corresponding author on reasonable request.
