# Supplementary material for: Clonal heterogeneity and rates of specific chromosome gains are risk predictors in childhood high‐hyperdiploid B‐cell acute lymphoblastic leukemia
Source: Mol Oncol. 2022 Jul 19;16(16):2899–919. doi: 10.1002/1878-0261.13276 (PMC9394234; doi:10.1002/1878-0261.13276)

a

	chr4	chr10	chr6	chrX	chr17	chr18	chr21	chr14
**Seq-iFISH**	0	0.38	0.38	0	4.15	1.04	1.50	3.96
**iFISH**	0.75	1.75	2	0	1.75	2.50	3.75	3.50

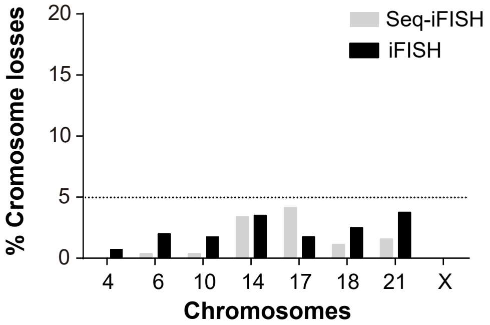

b

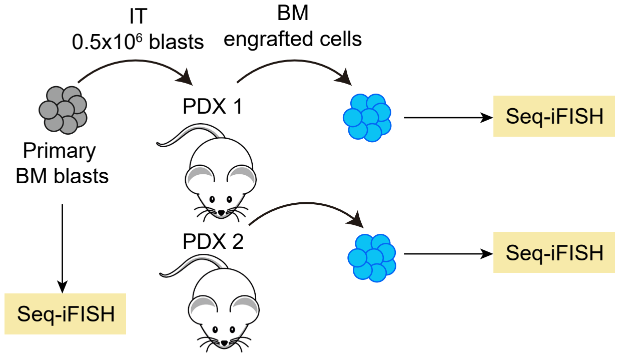

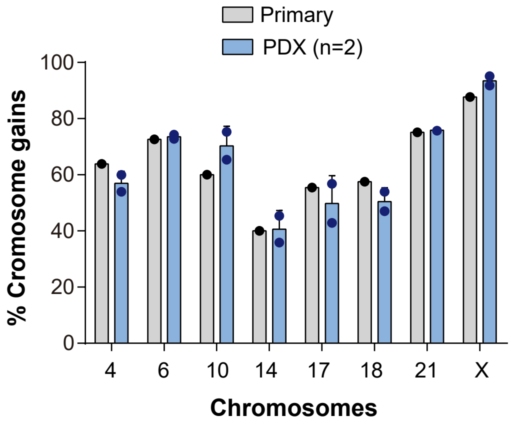

Supplementary FIGURE 2

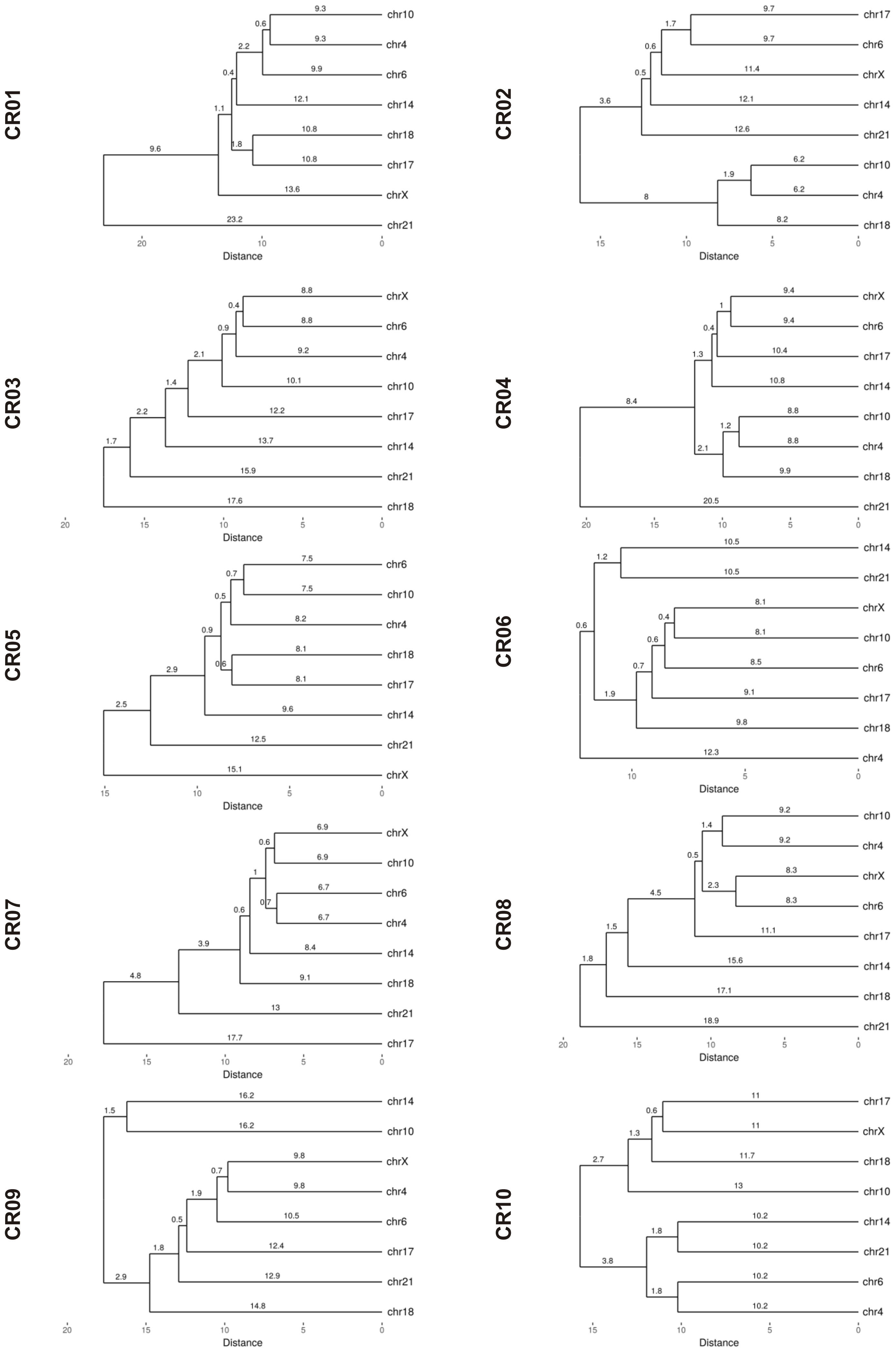

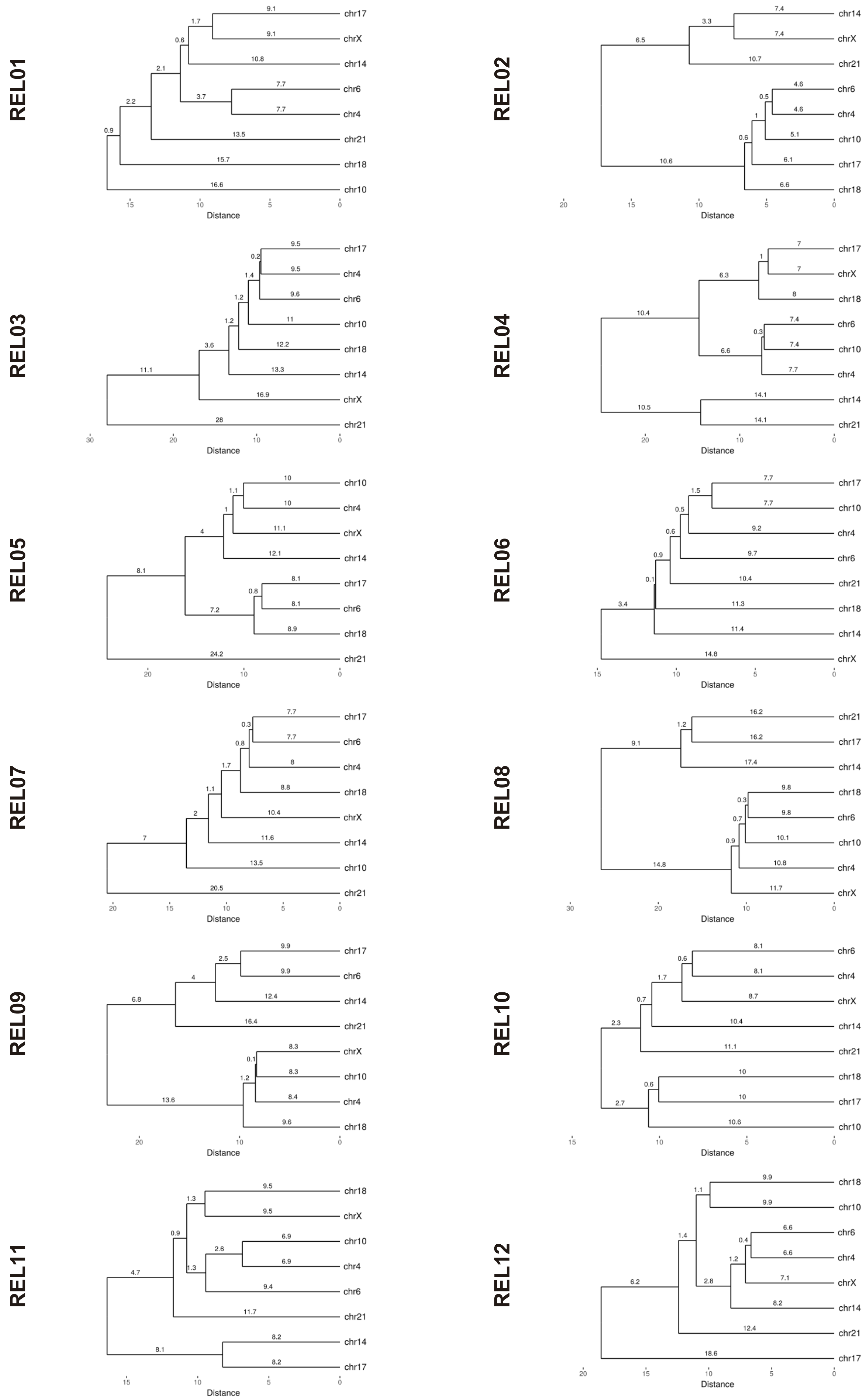
REL01
chr17
chrX
chr14
chr6
chr4
chr21
chr18
chr10
Distance
REL02
chr14
chrX
chr21
chr6
chr4
chr10
chr17
chr18
Distance
REL03
chr17
chr4
chr6
chr10
chr18
chr14
chrX
chr21
Distance
REL04
chr17
chrX
chr18
chr6
chr10
chr4
chr14
chr21
Distance
REL05
chr10
chr4
chrX
chr14
chr17
chr6
chr18
chr21
Distance
REL06
chr17
chr10
chr4
chr6
chr21
chr18
chr14
chrX
Distance
REL07
chr17
chr6
chr4
chr18
chrX
chr14
chr10
chr21
Distance
REL08
chr21
chr17
chr14
chr18
chr6
chr10
chr4
chrX
Distance
REL09
chr17
chr6
chr14
chr21
chrX
chr10
chr4
chr18
Distance
REL10
chr6
chr4
chrX
chr14
chr21
chr18
chr17
chr10
Distance
REL11
chr18
chrX
chr10
chr4
chr6
chr21
chr14
chr17
Distance
REL12
chr18
chr10
chr6
chr4
chrX
chr14
chr21
chr17
Distance

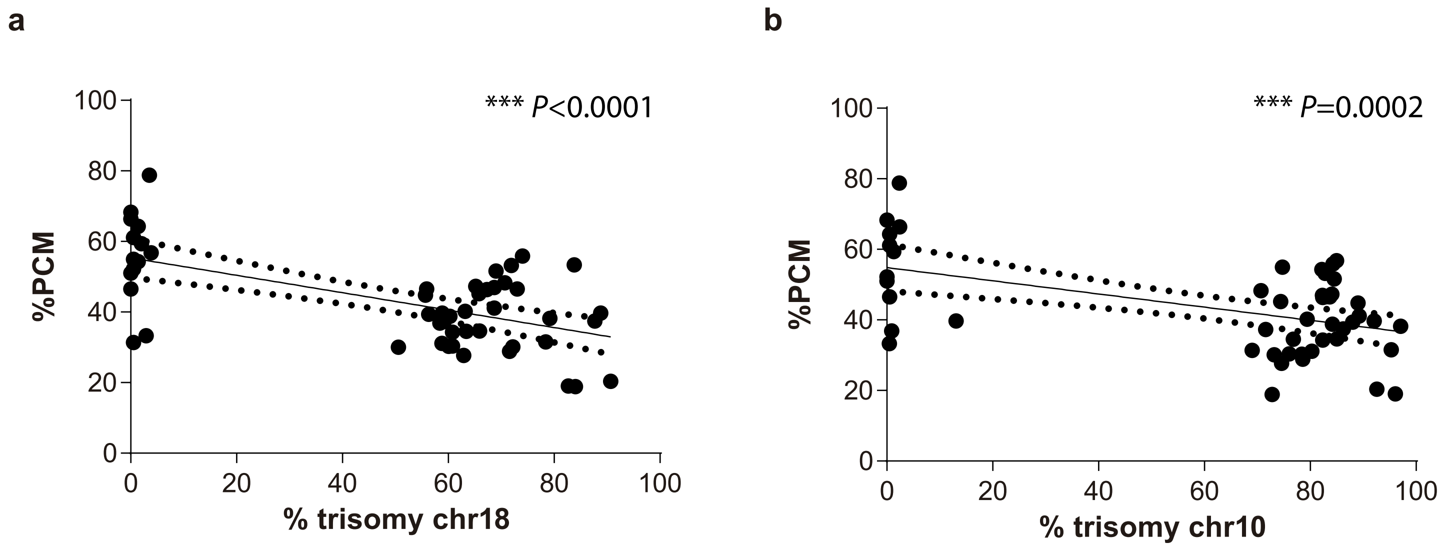
a
%PCM
100
80
60
40
20
0
0
20
40
60
80
100
% trisomy chr18
*** P<0.0001
b
%PCM
100
80
60
40
20
0
0
20
40
60
80
100
% trisomy chr10
*** P=0.0002

Supplementary FIGURE 5

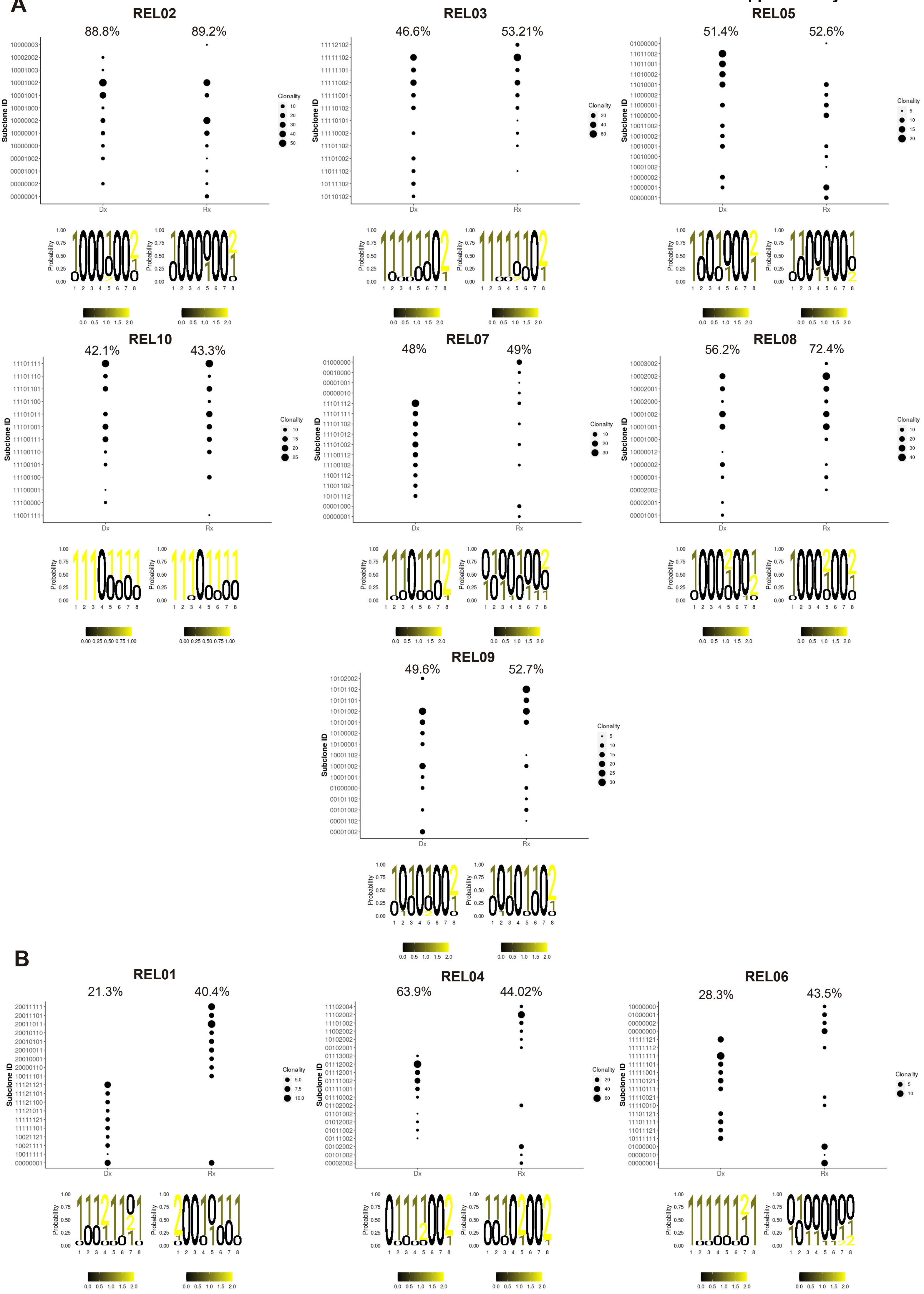

Supplement: Supplementary file 1 — Fig. S1. (Related to Fig. 2). Read‐out accuracy and reliability of Seq‐iFISH analyses. Fig. S2. (Related to Fig. 4). Hierarchical chromosomal gains in the indicated complete remission (CR) high‐hyperdiploid B‐cell acute lymphoblastic leukemia (HHD‐B‐ALL) patients. Fig. S3. (Related to Fig. 4). Hierarchical chromosomal gains in the indicated relapsed (REL) high‐hyperdiploid B‐cell acute lymphoblastic leukemia (HHD‐B‐ALL) patients. Fig. S4. (Related to Fig. 5). Aneuploidy levels are associated with chromosome instability (CIN). Fig. S5. (Related to Fig. 6). Individual longitudinal analysis of matched diagnostic‐relapse (DX‐REL) high‐hyperdiploid B‐cell acute lymphoblastic leukemia (HHD‐B‐ALL). [file MOL2-16-2899-s003.pdf]
