# Supplementary material for: Clonal heterogeneity and rates of specific chromosome gains are risk predictors in childhood high‐hyperdiploid B‐cell acute lymphoblastic leukemia
Source: Mol Oncol. 2022 Jul 19;16(16):2899–919. doi: 10.1002/1878-0261.13276 (PMC9394234; doi:10.1002/1878-0261.13276)
Supplement: Supplementary file 2 — Table S1. Cytogenetic and clinical data of all the childhood high‐hyperdiploid B‐cell acute lymphoblastic leukemia (HHD‐B‐ALL) samples used for blind validation analysis. [file MOL2-16-2899-s002.pdf]

CODE	Age	Gender	Karyotype/DI/FISH	WBC ($x10^9$/L)	Treatment protocol	MRD status post-ind*	Time to relapse (y)	DFS (y)	Death post-relapse	date of dx	date of rel	date last visit	chr gains (%)		% Major clone	Major clone	Clone	Predicted prognostic	Relapse (Yes/No)
													chr18	chr10					
HHD-01	2.4	F	55,XX,+4,+6,+8,+10,+14,+17,+18,der(19)t(?;19),+21[10]/55,XX,+4,+6,+8,+10,+17,+18,+21[2]/46,XX[3]	11	SHOP-2005	neg	-	9.7	-	13/03/2010	-	07/01/2020	71.6	78.6	28.9	433	+10, +18, +21, +21	FAVORABLE	NO
HHD-02	2.2	M	57,XY,+X,+4,+6,+9,+10,+14,+15,+17,+18,+21,+mar[20]/46,XY[5]	3.7	SHOP-2005	neg	-	12	-	07/04/2008	-	17/06/2020	60.1	78.4	30.3	433	+10, +18, +21, +21	FAVORABLE	NO
HHD-03	6.9	F	56,XX,+X,+1,der(1)del(1)(?p34)dup(1)(q21q42),dup(1)(q21q42), +4,+6,+10,+13,+14,+17,+18,+21[20]/46,XX[10]	9.1	PETHEMA LAL	neg	-	6.3	-	23/01/2014	-	17/06/2020	62.9	74.6	27.7	433	+10, +18, +21, +21	FAVORABLE	NO
HHD-04	4.7	F	61,XX,+X,+2,+4,+4,+5,+6,+8,+10,+12,+14,+15,+17,+17,+18,+21 [20]	15.9	SHOP-2005	neg	-	9.6	-	28/08/2010	-	19/05/2020	70.7	70.7	48.3	333	+10, +18, +21	FAVORABLE	NO
HHD-05	4	M	60,XXY,add(2)(q31),+4, +5,+5,+6,del(6)(q15q21), +8,+10,+11,del(11)(q13q21), +12,+14,+14,+17,+18, +21,+21[20]	226	PETHEMA LAL	neg	3		No	18/09/2010	03/04/2014	16/07/2020	56.3	88.0	39.4	433	+10, +18, +21, +21	FAVORABLE	YES
HHD-06	12	M	53,XY,+X,+8,+10,+14,i(10)(q10),+18,+21,+21[7]/53,XY,+X,idem,del(2)(q31)[3]/53,XY,+X,idem,t(6;12)(q15;q24)[4]/53,XY,+X,idem,add(12)(q24)[4]/46,XY[2]	2.7	PETHEMA LAL	1.6	-	10	-	21/09/2011	-	25/08/2020	60.3	84.2	38.8	433	+10, +18, +21, +21	FAVORABLE	NO
HHD-07	18	M	50~56,XY,+X,+4,+5,+6,+10,+14,+17,+18,+21,+21[12]/46,XY[2]	6.4	PETHEMA LAL	neg	2		Yes	25/01/2012	14/04/2014	23/05/2016	55.9	0.5	46.5	323	+18, +21	INFAVORABLE	YES
HHD-08	2	M	54~55,XY,+X,+4,+6,+8,+10,+17,+18,+21,+21[11]/46,XY[9]	115.3	PETHEMA LAL	neg	-	5	-	30/04/2012	-	02/08/2017	71.9	82.8	53.2	333	+10, +18, +21	FAVORABLE	NO
HHD-09	7	F	52,XX,+X,+X,+14,+21,+21,+mar[18]/46,XX[1]	6	PETHEMA LAL	neg	2		Yes	10/03/2009	10/09/2011	17/07/2020	0.0	0.0	51.0	322	+21	INFAVORABLE	YES
HHD-10	3	M	51-53,XXY,+9,+14,i(17)(q10),+19,+21[6]/46,XY[2]	8.7	PETHEMA LAL	neg	-	9	-	15/02/2011	-	03/03/2020	84.0	72.8	18.9	333	+10, +18, +21	FAVORABLE	NO
HHD-11	2	M	55,XX,der(1),+4,+6,+8,+8,+14,+18,+21,+21[6]/46,XX[14]	9.8	PETHEMA LAL	neg	-	10	-	03/08/2006	-	26/06/2017	65.2	84.1	47.3	333	+10, +18, +21	FAVORABLE	NO
HHD-12	2	M	53,XX,+6,+10,+14,+17,+18,+21,+21[11]/46,XX[9]	8.5	PETHEMA LAL	neg	-	8	-	05/07/2012	-	22/09/2020	59.2	71.6	37.3	333	+10, +18, +21	FAVORABLE	NO
HHD-13	8	M	56,XY,+X,+4,+6,+10,+10,+14,+17,+18,+21,+21[17]/56,XY,+X,dup(1)(q23q32),+4,+6,+10,+10,+14,+17,+18,+21,+21[2]/46,XY[4]	115	PETHEMA LAL	neg	-	7	-	27/07/2012	-	10/02/2020	78.4	95.3	31.5	443	+10, +10, +18, +21, +2	FAVORABLE	NO
HHD-14	5	M	60-61,XY[3]/46,XY[8]	6.6	PETHEMA LAL	neg	-	13	-	31/07/2006	-	11/12/2019	63.4	76.8	34.5	433	+10, +18, +21, +21	FAVORABLE	NO
HHD-15	4	M	50,XX,+1,del(1)(p22),+9,+21,+21[16]/50,XX,+1,del(1)(p22),+17,+21,+21,add(22)(p11)[3]/46,XX[1]	2.7	PETHEMA LAL	neg	1		No	05/03/2013	02/07/2014	20/03/2017	0.0	2.4	66.4	422	+21, +21	INFAVORABLE	YES
HHD-16	5	M	DI=1.14; FISH: Trisomies 4-10-17	8.86	PETHEMA LAL	neg	-	9	-	24/10/2011	-	18/11/2020	72.2	73.2	30.1	333	+10, +18, +21	FAVORABLE	NO
HHD-17	2	M	60-65,XY,+2,+5,+6,+8,+10,+11,+14,+15,+16,+17,+18,+21,+21,+22[7]/46,XY[9]	3.7	PETHEMA LAL	neg	-	9	-	28/02/2011	-	13/07/2020	79.2	97.1	38.2	333	+10, +18, +21	FAVORABLE	NO
HHD-18	1	F	DI= 1.17 (13% blasts); FISH: aneuplody	65.14	PETHEMA LAL	neg	-	8	-	17/01/2012	-	17/09/2020	90.7	92.6	20.4	466	+10, +10, +10, +18, +18, +18, +21, +21	FAVORABLE	NO
HHD-19	3	M	49~56,XY,+X,+Y,+4,+6,+7?,+8?,+10,+14,+19,+21,+21[11]/46,XY[9]	2	PETHEMA LAL	neg	-	7	-	16/10/2012	-	14/10/2020	0.5	69.0	31.4	432	+10, +21, +21	INFAVORABLE	NO
HHD-20	5	M	55,XY,+X,dup(1)(q22q42),+4,+5,+6,del(6)(q21q25),+10,+14,+17,+21,+21[17]/46,XY[3]	6.1	PETHEMA LAL	neg	1		Yes	04/10/2011	12/09/2012	31/12/2014	0.0	83.7	46.5	432	+10, +21, +21	INFAVORABLE	YES
HHD-21	9	M	DI hyperdiploidy; FISH: Trisomies 4-10-17	1.65	PETHEMA LAL	neg	-	8	-	16/04/2012	-	22/10/2020	58.8	80.3	31.1	333	+10, +18, +21	FAVORABLE	NO
HHD-22	5	M	53,XX,+X,+4,der(4),+6,del(9)(p11),t(10;13)(q26;q12),+14,-13,add(16)(q23),i(17)(q10),+21,+21,+marx2[20]/46,XX[2]	88.3	PETHEMA LAL	neg	-	7	-	31/12/2012	-	16/11/2020	2.0	1.3	59.4	322	+21	INFAVORABLE	NO
HHD-23	3	F	53,XX,dup(1),+4,+6,+14,+15,+17,+18,+21,+22[7]/46,XX[6]	33.9	PETHEMA LAL	neg	-	12	-	06/05/2008	-	14/07/2020	50.6	73.2	30.0	432	+10, +21, +21	FAVORABLE	NO
HHD-24	1	M	52,XY,+X,+6,+14,+17,+21,+21[17]/46,XY[3]	61530	AIEOP-BFM ALL	0.0093	1.2	1.2	No	20/02/2017	04/05/2018	19/12/2018	0.5	0.5	61.1	322	+21	INFAVORABLE	YES
HHD-25	1	M	52~53,XY,+Y,inc[4]/46,XY[16]	9600	AIEOP-BFM ALL	0.0063	2.1	2.1	No	19/02/2015	26/03/2017	29/01/2019	1.4	0.5	64.3	322	+21	INFAVORABLE	YES
HHD-26	3	M	53,XY,inc[12]	22060	AIEOP-BFM ALL	neg	1.6	1.6	Yes	19/11/2010	25/06/2012	21/09/2013	0.5	0.0	52.2	322	+21	INFAVORABLE	YES
HHD-27	5	M	na	5850	AIEOP-BFM ALL	neg	-	6	-	29/10/2014	-	13/11/2018	58.4	0.9	36.9	323	+18, +21	INFAVORABLE	NO
HHD-28	2	M	60,XY,t(1;?)(q?;?),+21,+22,inc[6]/46,XY[5]	13890	AIEOP-BFM ALL	neg	-	5.1	-	27/06/2013	-	19/07/2018	63.2	79.4	40.2	333	+10, +18, +21	FAVORABLE	NO
HHD-29	7	M	55,XY,+X,+?5,+6,+9,+10,+d,+18,+21,+21[24]/46,XY[16]	2230	AIEOP-BFM ALL	neg	-	7	-	03/10/2013	-	04/10/2018	65.9	85.0	34.6	333	+10, +18, +21	FAVORABLE	NO
HHD-30	1	M	51-53,XY,inc[4]/46,XY[7]	42300	AIEOP-BFM ALL	neg	3.8	3.8	No	20/01/2012	07/11/2015	05/12/2018	0.0	0.0	68.3	322	+21	INFAVORABLE	YES
HHD-31	4	M	DI=1 ,20 ; FISH: +4, +10, +17, +21	2.68	PETHEMA LAL	neg		6	-	17/02/2015	-	11/03/2020	83.8	82.8	53.4	333	+10, +18, +21	FAVORABLE	NO
HHD-32	5	M	54,XY,+X,+b,+6,+10,+?16,+18,+21,+?21[7]/46,XY[8]	14820	AIEOP-BFM ALL	neg	3.5		No	05/09/2011	05/03/2015	08/11/2018	65.8	74.4	45.2	333	+10, +18, +21	FAVORABLE	YES
HHD-33	2	M	na	9800	AIEOP-BFM ALL	neg	4.2	12	No	26/02/2009	-	24/04/2013	74.0	84.3	55.9	333	+10, +18, +21	FAVORABLE	NO
HHD-34	1	F	57-59,XX,+4,+5,+6,inc[5]/46,XX[7]	12980	AIEOP-BFM ALL	neg	-	4	No	28/11/2014	-	30/11/2018	69.0	84.5	51.6	333	+10, +18, +21	FAVORABLE	NO
HHD-35	5	M	56,XY,inc[4]/46,XY[10]	18480	AIEOP-BFM ALL	neg	2.3		No	14/12/2012	02/04/2015	16/11/2018	60.83	76.04	30.41	333	+10, +18, +21	FAVORABLE	YES
HHD-36	3	F	57,XX,+X,add(4)(q34),+6,+?8,+10,+14,+?16,+18,+?20,+21,+21,inc[20]	10190	AIEOP-BFM ALL	neg	5	8	No	25/01/2013	-	25/01/2018	88.79	92.06	39.72	333	+10, +18, +21	FAVORABLE	NO
HHD-37	10	F	55,XX,+X,+X,+4,+6,+10,+17,+18,+21,+21[5]/55,XX,idem,t(8;?)(p21;?),12q-[3]/46,XX[21]	1.45	SHOP-2005	neg		8	No	15/10/2012	15/06/2015	17/04/2020	60.78	82.35	34.31	333	+10, +18, +21	FAVORABLE	YES
HHD-38	3	M	53,XY,+X,+4,+5,+6,+14,+16,+17[2]/46,XY[30]	4.86	SHOP-2005	neg	2.4	10	No	28/04/2008	10/08/2008	15/06/2020	3.53	2.35	78.82	322	+21	INFAVORABLE	YES
HHD-39	3	M	53,XY,+X,+6,+10,+14,+18,+21,+mar/46,XY	124.95	SHOP-2005	neg	-	9	-	26/11/2010	-	19/12/2019	67.32	82.35	46.41	333	+10, +18, +21	FAVORABLE	NO
HHD-40	5	F	56,XX,+4,+6,+7,+8,+14,+17,+18,+21,+21,+22	7.51	SHOP-2005	neg	-	13	-	29/08/2007	-	14/12/2020	58.88	13.08	39.72	323	+18, +21	INFAVORABLE	NO
HHD-41	1	M	57-58,XY,+4,+5,+6,+9,+10,+17,+18,+19,+21,+3mar[4]//46,XY[65]	4.57	SHOP-2005	neg	-	9	-	08/07/2011	-	21/10/2019	68.71	82.31	46.94	333	+10, +18, +21	FAVORABLE	NO
HHD-42	2.3	M	56,XXY,+3,+4,+6,+10,+14,+18,+21,+21[5]/46,XY[15]	25.4	SHOP-2005	neg	-	8.8	-	22/10/2010	-	23/09/2019	68.69	89.25	41.12	333	+10, +18, +21	FAVORABLE	NO
HHD-43	1	F	57,XX,+X,+4,+6,+10,+14,+14,+17,+18,+18,+21,+21[19]/46,XX[9]	14.2	PETHEMA LAL	neg	-	6.8	-	27/08/2013	-	14/07/2020	87.68	86.26	37.44	333	+10, +18, +21	FAVORABLE	NO
HHD-44	3	F	58,XXXX,+4,+4,+5,idic(7)(q11.1),+8,+10,+13,+17,+18,+21,+21[9]/58,XXXX,+4,+4,+5,+8,+10,+13,+17,+18,+21,+21[11]	9.7	SHOP-2005	neg	-	6.7	-	18/09/2012	-	24/07/2019	55.71	89.05	44.76	333	+10, +18, +21	FAVORABLE	NO
HHD-45	2	M	High hyperdiploid by FISH	11.9	UKALL2003	0.05	1.8	1.8	Yes	10/08/2010	25/05/2012	01/01/2013	0.50	74.75	54.95	332	+10, +21	INFAVORABLE	YES
HHD-46	6	M	na	35080	AIEOP-BFM ALL	neg	4.3		No	04/08/2008	20/11/2012	08/05/2013	2.94	0.49	33.33	422	+21, +21	INFAVORABLE	YES
HHD-47	6	M	67,XY,+Y,t(1;?)(q?;?),+3,?del(3)(p21),+21,inc[13]/46,XY[7]	8500	AIEOP-BFM ALL	0.001	-	4.4	-	09/09/2014	-	21/01/2019	82.68	96.10	19.05	333	+10, +18, +21	FAVORABLE	NO
HHD-48	1	M	56~58,X,+X,+X,-Y,+4,+5,+6,+8,+10,+14,+14,+18,+20,+21,+21[8]/46,XY[12]	4940	AIEOP-BFM ALL	0.0013	2.2		No	22/09/2014	03/12/2016	17/01/2019	3.88	84.95	56.80	332	+10, +21	INFAVORABLE	YES
HHD-49	1	M	61-63,XY,+Y,+3,+4,+5,+6,+8,+10,+?10,+?12,+14,+?14,+17,+21,+21,+22,+2mar[cp7]/46,XY[14]	3370	AIEOP-BFM ALL	neg	-	4.1	-	13/11/2014	-	13/12/2018	73.02	82.79	46.51	333	+10, +18, +21	FAVORABLE	NO
HHD-50	14	M	53,XY,+X,+Y,+4,+6,-7,+10,+11,- 13,+14,+21,+21[5]/53,XY,+Y,idem,add(16)(q24),+17[15]	5.19	PETHEMA LAL	4.7	-	-	No	25/09/2018	21/11/2019	18/09/2020	1.44	82.21	54.33	432	+10, +21, +21	INFAVORABLE	YES

*MRD measured by flow cytometry or Ig clonotype detection

Supplementary Table 1: Cytogenetic and clinical data of all the childhood HHD-B-ALL samples used for blind validation analysis.
