# Supplementary material for: Clonal heterogeneity and rates of specific chromosome gains are risk predictors in childhood high‐hyperdiploid B‐cell acute lymphoblastic leukemia
Source: Mol Oncol. 2022 Jul 19;16(16):2899–919. doi: 10.1002/1878-0261.13276 (PMC9394234; doi:10.1002/1878-0261.13276)
Supplement: Supplementary file 3 — Table S2. Levels of ‘false’ gains and losses observed by Seq‐iFISH analysis in the indicated control (Ctrl) samples. [file MOL2-16-2899-s001.pdf]

Ctrl	Sample	Karyotype	Anomaly	Chr4	chr6	chr10	chr14	chr17	chr18	chr21	chrX
1	FL-CD34+	46, XX	gains	0	0	0	0	0	0	0	0
			losses	0	2	1.33333333	2	2	2	0.66666667	0
2	FL-CD34+	46 XY	gains	0	0	0	0.37735849	0.37735849	0.37735849	0.37735849	0.37735849
			losses	0	0.37735849	0.37735849	3.39622642	4.1509434	1.13207547	1.50943396	0
3	PB	46, XX	gains	0	0	0	0	0	0	0	0
			losses	0.52356021	1.57068063	1.04712042	5.23560209	3.14136126	1.04712042	1.57068063	1.04712042
**Average**			gains	0	0	0	0.12578616	0.12578616	0.12578616	0.12578616	0.12578616
			losses	0.17452007	1.31601304	0.91927075	3.54394284	3.09743488	1.3930653	1.24892709	0.34904014
