# Supplementary material for: Clonal heterogeneity and rates of specific chromosome gains are risk predictors in childhood high‐hyperdiploid B‐cell acute lymphoblastic leukemia
Source: Mol Oncol. 2022 Jul 19;16(16):2899–919. doi: 10.1002/1878-0261.13276 (PMC9394234; doi:10.1002/1878-0261.13276)
Supplement: Supplementary file 4 [file MOL2-16-2899-s004.docx]

**Supplementary Table 1: Cytogenetic and clinical data of all the childhood high-hyperdiploid B-cell acute lymphoblastic leukemia (HHD-B-ALL) samples used for blind validation analysis**. *minimal residual disease (MRD) measured by flow cytometry or Ig clonotype detection. F: female, M: male, WBC: white blood cell count, DFS: disease free survival, dx: diagnosis, rel: relapse, chr: chromosome.

**Supplementary Table 2: Levels of “false” gains and losses observed by Seq-iFISH analysis in the indicated control (Ctrl) samples.** Rates of chromosome gains and losses observed by Seq-iFISH analyses in the indicated samples. Average values for the three independent analyses are provided (bottom). FL: fetal liver, PB: peripheral blood, chr: chromosome.

**Supplementary Figure 1 (Related to Figure 2). Read-out accuracy and reliability of Seq-iFISH analyses.** (**a**) Comparison of results obtained by Seq-iFISH and single-hybridization iFISH experiments with the indicated chromosomes in Ctrl#2. (**b**) Seq-iFISH results of CR01 primary sample and two primary-derived xenograft (PDX) samples.

**Supplementary Figure 2 (Related to Figure 4).** Hierarchical chromosomal gains in the indicated complete remission (CR) high-hyperdiploid B-cell acute lymphoblastic leukemia (HHD-B-ALL) patients.

**Supplementary Figure 3 (Related to Figure 4).** Hierarchical chromosomal gains in the indicated relapsed (REL) high-hyperdiploid B-cell acute lymphoblastic leukemia (HHD-B-ALL) patients.

**Supplementary Figure 4 (Related to Figure 5). Aneuploidy levels are associated with chromosome instability (CIN).** The frequencies of trisomies 18 (*left*) and 10 (*right*) correlate with the percentage of major clone (PCM) obtained from the blind validation iFISH analysis on an independent patient cohort (n=50).

**Supplementary Figure 5 (Related to Figure 6). Individual longitudinal analysis of matched diagnostic-relapse (DX-REL) high-hyperdiploid B-cell acute lymphoblastic leukemia (HHD-B-ALL)**. Longitudinal analysis of matched DX-REL HHD-B-ALL individual samples showing the major leukemic clones observed in matched DX and REL samples, being either shared (**A**) or distinct (**B**).
